# Supplementary material for: Low-voltage ultrafast nonvolatile memory via direct charge injection through a threshold resistive-switching layer
Source: Nat Commun. 2022 Aug 6;13:4591. doi: 10.1038/s41467-022-32380-3 (PMC9357017; doi:10.1038/s41467-022-32380-3)
Supplement: Supplementary file 1 — Supplementary Information [file 41467_2022_32380_MOESM1_ESM.pdf]

Supplementary Information for

Low-voltage ultrafast nonvolatile memory via direct charge injection through a threshold resistive-switching layer

Yuan Li[1,#], Zhi Cheng Zhang[1,#], Jiaqiang Li[2,3], Xu-Dong Chen[1,*], Ya Kong[2], Fu-Dong Wang[1], Guo-Xin Zhang[1], Tong-Bu Lu[1,*] & Jin Zhang[2,*]

[1]MOE International Joint Laboratory of Materials Microstructure, Institute for New Energy Materials and Low Carbon Technologies, School of Material Science and Engineering, Tianjin University of Technology, Tianjin 300384, China.
[2]Center for Nanochemistry, Beijing Science and Engineering Center for Nanocarbons, Beijing National Laboratory for Molecular Sciences, College of Chemistry and Molecular Engineering, Peking University, Beijing 100871, China.
[3]Advanced Membranes and Porous Materials Center, Physical Sciences and Engineering Division, King Abdullah University of Science and Technology, Thuwal 23955-6900, Saudi Arabia.
[#]These authors contributed equally: Yuan Li, Zhi-Cheng Zhang.
*E-mail: chenxd@email.tjut.edu.cn; lutongbu@tjut.edu.cn; jinzhang@pku.edu.cn.

Experimental Details

1. Preparation of GDYO

Preparation of hexaethynylbenzene (HEB) monomer

Hexakis[(trimethylsilyl)ethynyl]benzene (HEB-TMS) was first synthesized according to the synthetic route presented in Supplementary Fig. 1[1]. Then 5 mg HEB-TMS monomers were dissolved into 75 mL CH_2Cl_2 solvent in a Schlenk reactor. 75 μL solution of tetrabutylammonium fluoride (1 M in tetrahydrofuran) was added into the HEB-TMS solution. After reaction at 0 °C for 15 min under Ar atmosphere in the dark, HEB solution (in CH_2Cl_2) was obtained as the precursor for the subsequent coupling reaction.

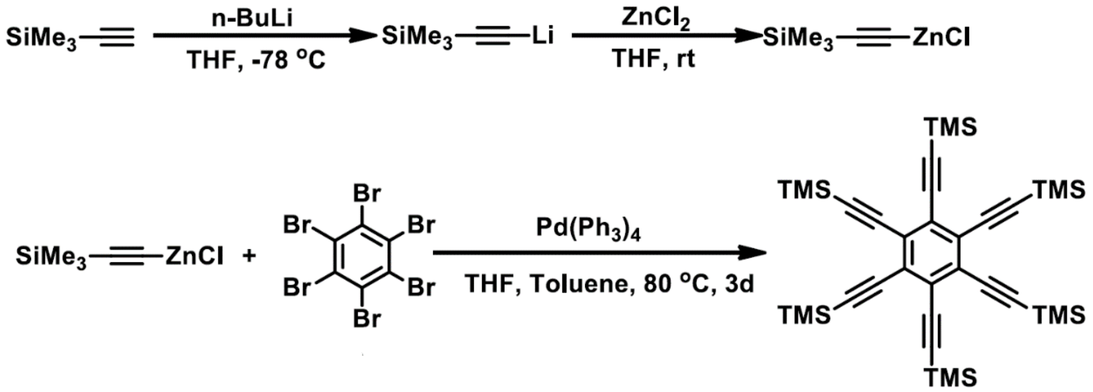

Supplementary Fig. 1| Synthetic route for the HEB-TMS monomers.

Synthesis of GDY on Cu foil

The GDY film was synthesized via an electric-double-layer (EDL)-confined strategy in a routine two-electrode cell (Supplementary Fig. 2a)[2]. Here an Au foil, a Cu foil, and 50 mL acetone were used as the anode, cathode and electrolyte, respectively. Then, 0.25 mL N,N,N',N'-tetramethylethylenediamine (TMEDA) and 0.2 mL HEB monomer solution were introduced into the cell. The voltage between two electrodes was set as 2 V. After 10 h successive reaction in dark environment at 20 °C, uniform GDY film was synthesized on Cu foil (Supplementary Fig. 2b). Finally, the sample was washed in turn with acetone, dimethylformamide (DMF), ethanol and water, followed by N_2-flow drying.

Preparation of GDYO film

The GDY film on Cu foil was first transferred onto SiO_2/Si substrate (Supplementary Fig. 2c) via a poly (methyl methacrylate) (PMMA)-assisted "electrochemical bubbling" method[3]. Then the GDY film was treated by an UV-ozone cleaner (200 W) for 120 s to form GDYO film (Supplementary Fig. 2d).

Supplementary Figs. 3 and 5 depict the X-ray photoelectron spectroscope (XPS) and Raman spectra of the GDY and GDYO films, respectively. For the GDY film before oxidation, its O 1*s* peak can be deconvoluted into three subpeaks corresponding to Si–O, C–O and C=O bonds (Supplementary Fig. 3c), and the Si–O bond from SiO_2 substrate is dominant. After UV-ozone treatment, a new subpeak corresponding to O=C–OH bond appears, and the proportion of C=O bonds is significantly increased (Supplementary Fig. 3d). Similarly, an obvious increase of subpeaks for the O=C–OH and C=O bonds are also observed in the C 1s peak as illustrated in Supplementary Figs. 3e,f. These results demonstrate the oxidation of GDY film via UV-ozone treatment. As shown in Supplementary Figs. 3e,f, the ratio of the subpeaks for C≡C and C=C decreases from 1.4 to 0.8 after UV-ozone treatment, which indicates that C≡C with higher activity is more likely to be oxidized. This phenomenon was also demonstrated by the Raman spectra. As shown in Supplementary Fig. 5, the Raman bands at 1926 cm^{-1} and 2174 cm^{-1} corresponding to the vibration of C≡C bond[1] are almost disappeared after UV-ozone treatment.

To analyze the trace elements induced during the synthesis of GDY, the XPS of GDY film before and after oxidation were also measured on sapphire substrate, using a bare sapphire as reference. From the survey scans as shown in Supplementary Figs. 4a–c, we observed weak peaks corresponding to Si and Cu elements. Supplementary Figs. 4d–f present the high-resolution Si 2*p* peaks of bare sapphire, GDY and GDYO films on sapphire. Noteworthily, a weak peak of Si 2*p* (98.3 eV) was observed even on bare sapphire which might originate from impurities in sapphire. For the GDY and GDYO films, a new subpeak appears at 102.4 eV, indicating that trace Si was induced to the GDY from the HEB-TMS monomers during the synthesis process. Similarly, the appearance of a weak Cu 2*p* peak demonstrates that GDY and GDYO films contain trace Cu induced from the Cu substrate during the synthesis process (Supplementary Figs. 4g–i).

Fourier transform infrared spectroscope (FTIR) was also used to characterize the GDY before and after oxidation (Supplementary Fig. 6). For the GDY film, the bands located at 1627 cm^{-1} is attributed to the skeletal vibration of benzene ring, while the wide band at 2107 cm^{-1} is due to the stretching vibration of C≡C bond[4]. In comparison, the GDYO film after UV-ozone treatment has an enhanced band at 1103 cm^{-1} and 1727 cm^{-1}, which are ascribed to the stretching vibration of C–O and C=O bonds[5,6], and the band corresponding to C≡C almost disappears. The band located around 619 cm^{-1} is attributed to the bending vibration of O–H[5]. These results suggest that a large amount of oxygen-containing groups are bonded to GDY by UV-ozone treatment, and the *sp*-hybrid carbon atoms (C≡C) are the main oxidation sites.

Supplementary Fig. 7 depicts the UV-vis-NIR absorption spectra of GDY and GDYO films, and the corresponding band gaps of GDY and GDYO obtained from the Tauc plots are 2.40 eV and 4.14 eV, respectively. Furthermore, the ultraviolet photoelectron spectroscopy (UPS) measurement was performed to describe the band diagrams of GDY and GDYO films. As shown in Supplementary Fig. 8, the conduction band and valence band of GDY and GDYO are 4.00 eV (GDY), 2.94 eV (GDYO) and 6.40 eV (GDY), 7.08 eV (GDYO), respectively. Thus the band gap of GYDO is significantly enlarged in comparison with that of GDY.

Since numerous oxygen-containing groups were introduced into the GDYO film, the conductivity of GDYO degrades dramatically in comparison with that of GDY film. The output curves of a lateral GDY device were measured before and after UV-ozone treatment. As shown in Supplementary Fig. 9, the GDYO film after treatment is almost non-conductive, and its conductance is several orders of magnitude smaller than that of the GDY film before treatment.

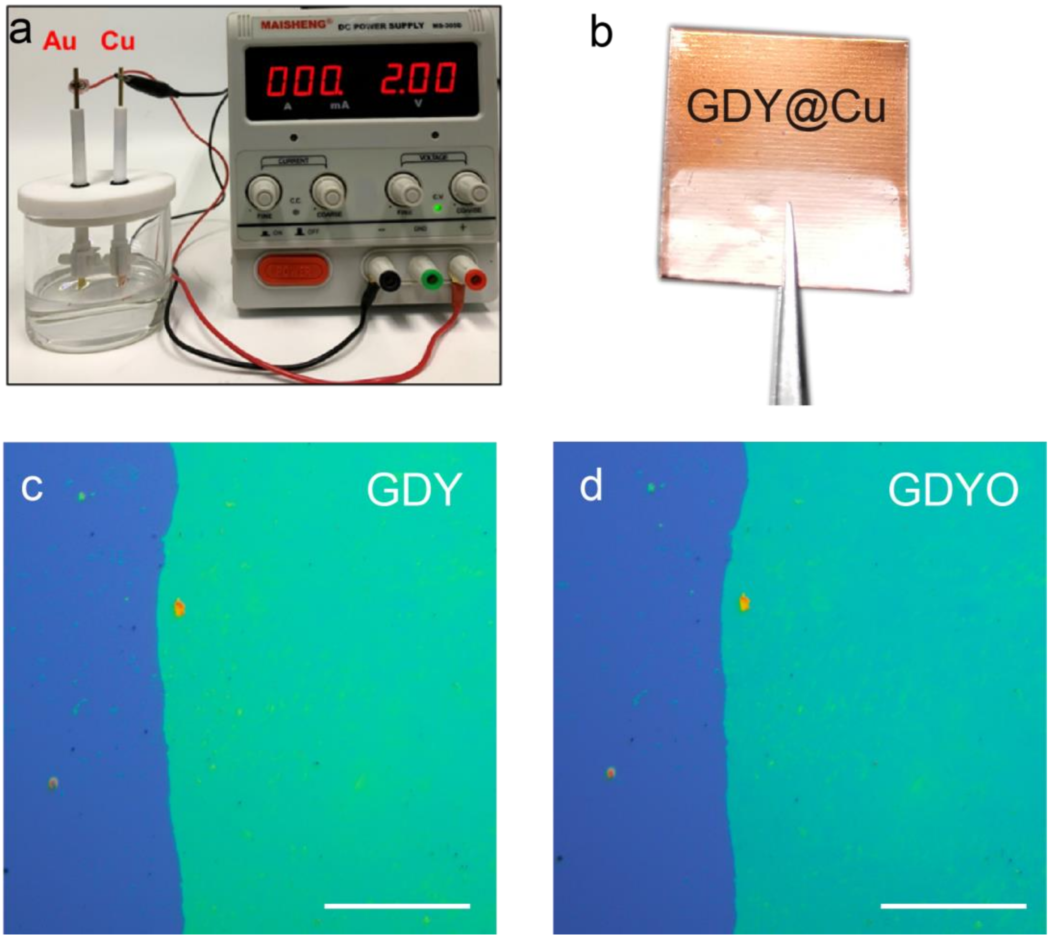

Supplementary Fig. 2| Preparation of GDYO film. **a**, Experimental setup of the EDL-confined method for the synthesis of GDY film. **b**, Photograph of the synthesized GDY film on Cu foil. **c**, Optical microscope (OM) image of the GDY film transferred on a SiO_2/Si substrate. **d**, OM image of the GDYO film prepared via UV-ozone treatment. Scale bars, 50 μm.

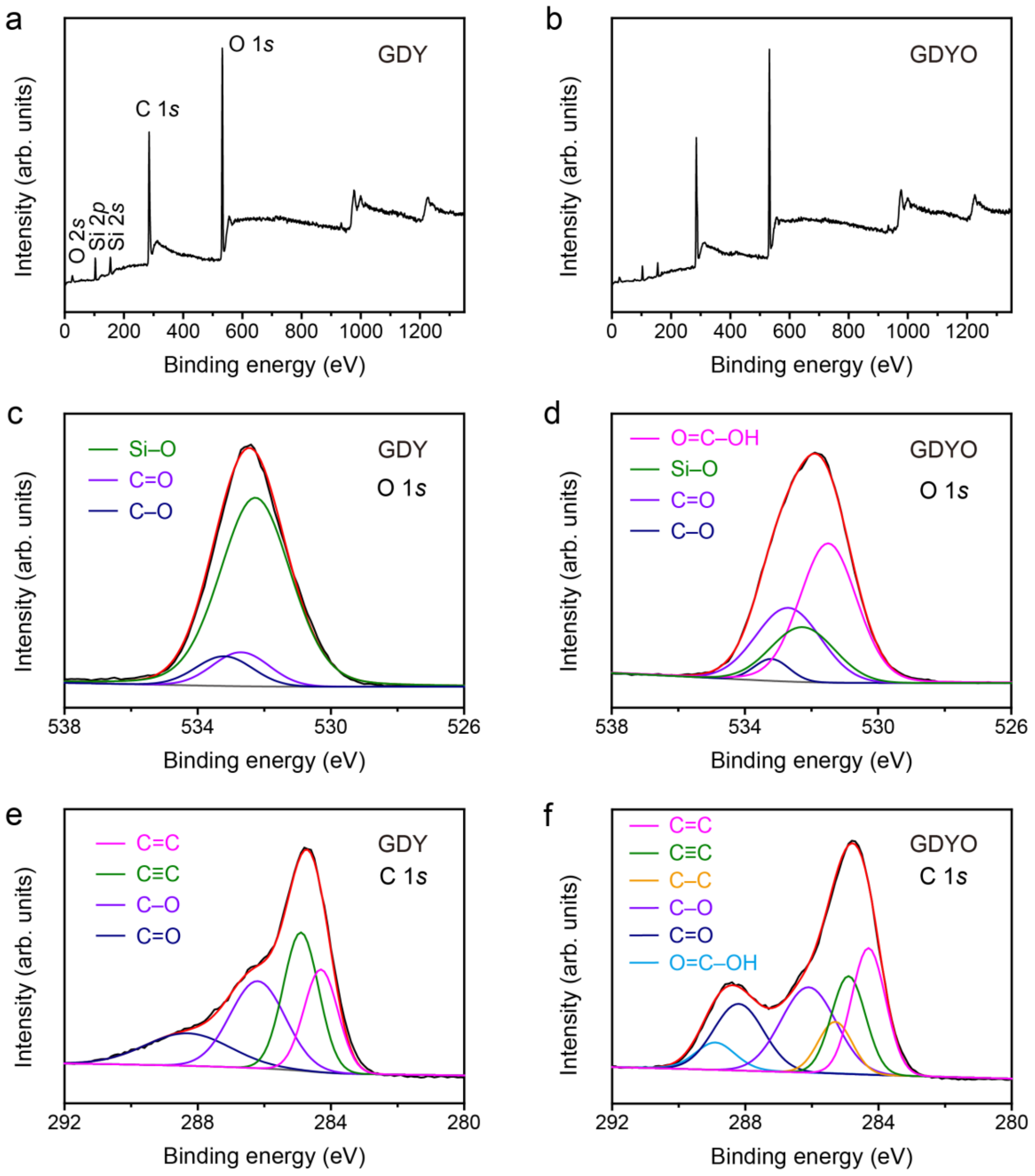

Supplementary Fig. 3| XPS of GDY and GDYO films on SiO_2/Si substrate. **a**,**b**, Survey scans of the GDY (**a**) and GDYO films (**b**) on SiO_2/Si substrate. **c**,**d**, High resolution XPS of O 1*s* peak for GDY (**c**) and GDYO films (**d**), respectively. **e**,**f**, High resolution XPS of C 1*s* peak for GDY (**e**) and GDYO films (**f**), respectively.

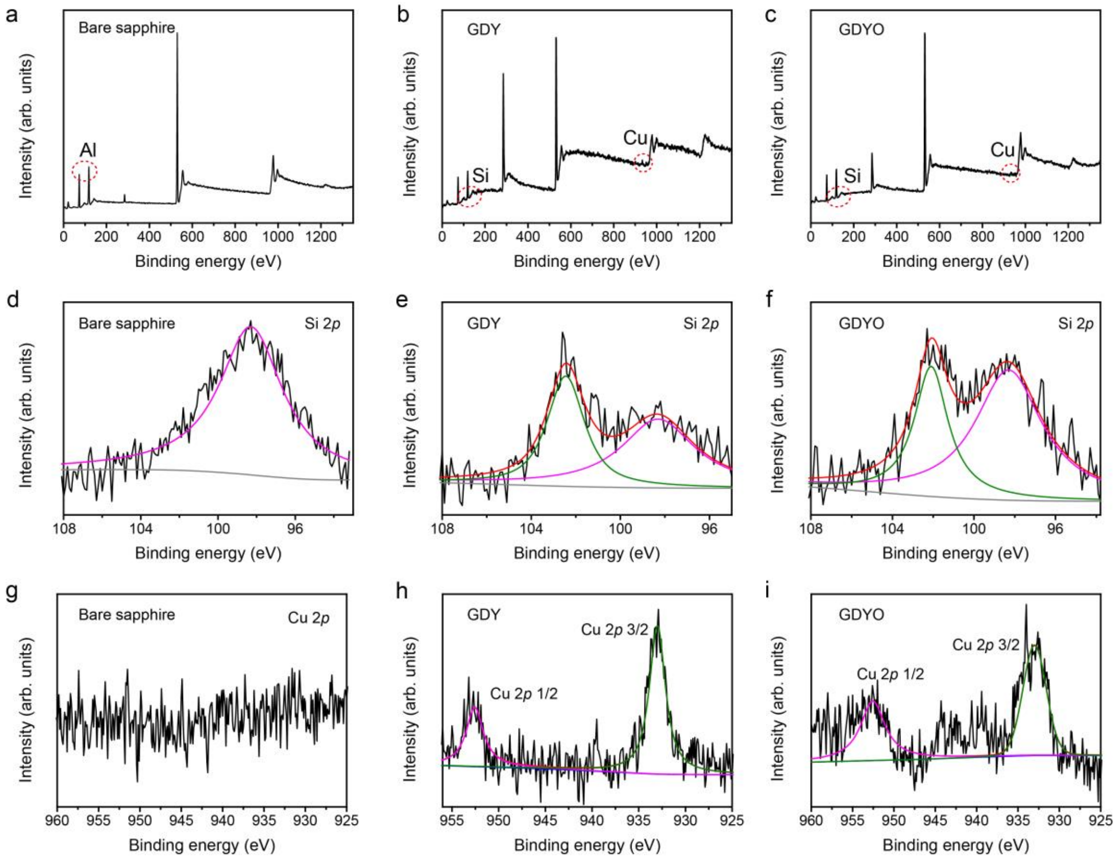

Supplementary Fig. 4| XPS of GDY and GDYO films on sapphire substrate. **a**–**c**, Survey scans of bare sapphire (**a**), GDY (**b**) and GDYO films (**c**) on sapphire substrate, respectively. **d**–**f**, High resolution XPS of Si 2*p* peak for bare sapphire (**d**), GDY (**e**) and GDYO films (**f**) on sapphire substrate, respectively. **g**–**i**, High resolution XPS of Cu 2*p* peak for bare sapphire (**g**), GDY (**h**) and GDYO films (**i**) on sapphire substrate, respectively.

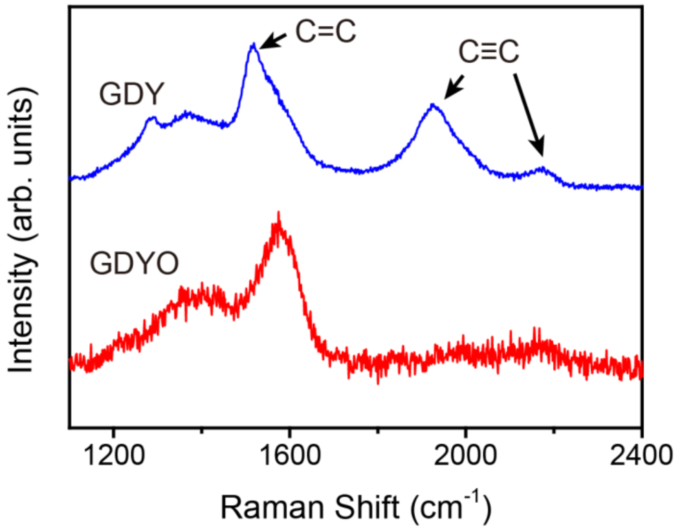

Supplementary Fig. 5| Raman spectra of GDY (blue) and GDYO (red) films.

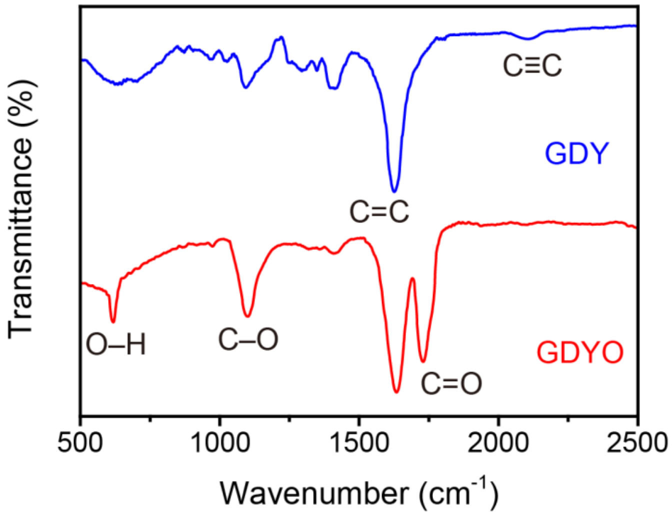

Supplementary Fig. 6| FTIR spectra of GDY (blue) and GDYO (red).

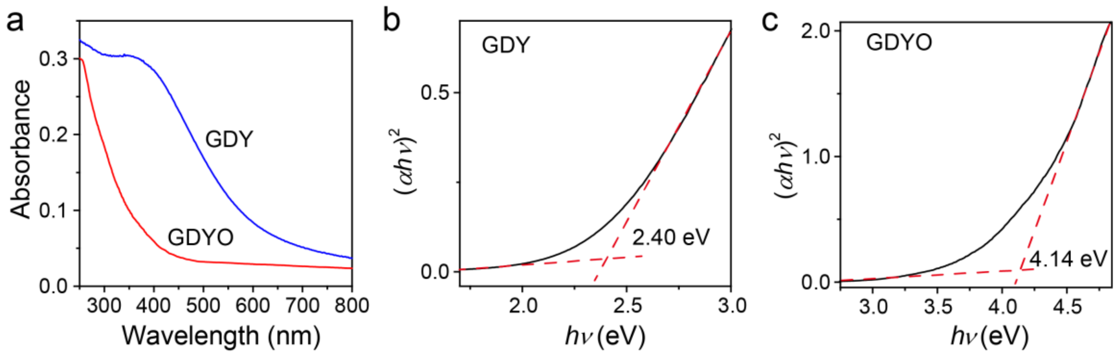

Supplementary Fig. 7| Absorbance spectra and Tauc plots of GDY and GDYO. **a**, UV-vis-NIR absorbance spectra of GDY and GDYO. **b**,**c**, Corresponding Tauc plots of GDY (**b**) and GDYO (**c**) films. The band gaps of GDY and GDYO extracted from the

Tauc plots are 2.40 eV and 4.14 eV, respectively.

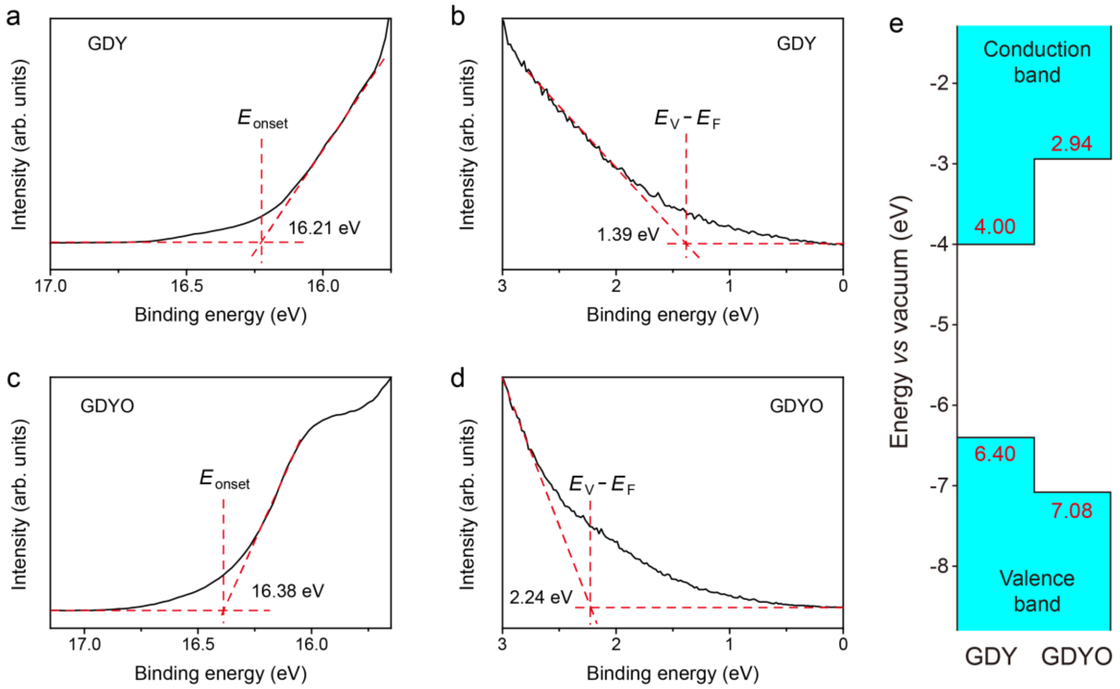

Supplementary Fig. 8| Band structure of GDY and GDYO measured by UPS spectra. **a**–**d**, UPS spectra of GDY (**a**,**b**) and GDYO (**c**,**d**) films, respectively. **e**, Band diagrams of GDY and GDYO. The Fermi level can be calculated using the equation $E_F = h\upsilon - E_{onset}$, where $h\upsilon$ is the incident photon energy (21.22 eV), and E_{onset} is the onset level related to the secondary electrons[7]. Thus the Fermi levels of GDY and GDYO are 5.01 eV and 4.84 eV, respectively. The cutoff of the lowest binding energy (as illustrated in **b** and **d**) indicates the difference between the energy of Fermi level and valence band maximum ($E_V - E_F$)[7]. and thus the valence bands of GDY and GDYO are calculated as 6.40 eV and 7.08 eV, respectively. Combing the optical band gaps obtained from the Tauc plots as shown in Supplementary Fig. 6, which are 2.40 eV (GDY) and 4.14 eV (GDYO), the conduction bands of GDY and GDYO are 4.00 eV and 2.94 eV, respectively. Thus we can obtain the band diagrams of GDY and GDYO as illustrated in **e**.

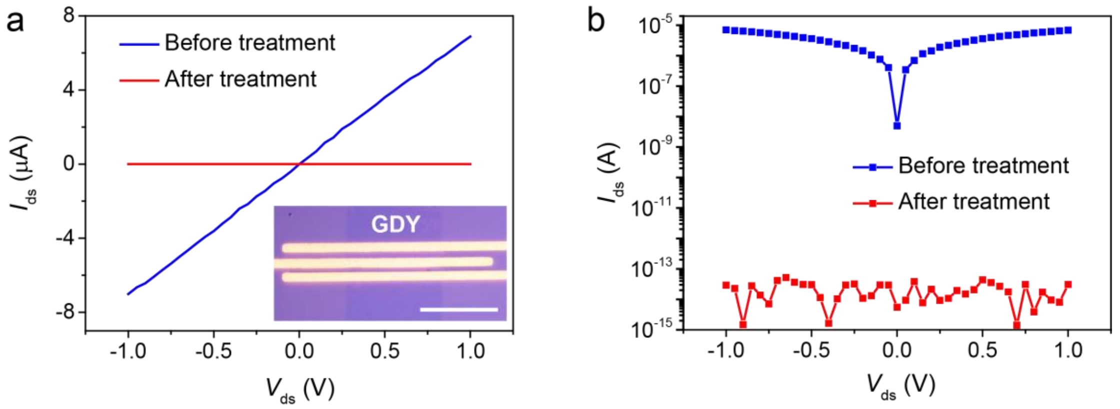

Supplementary Fig. 9| Output curves of a lateral GDY device before and after UV-ozone treatment. The ordinate in **a** and **b** are in the form of liner (**a**) and logarithmic (**b**) coordinates, respectively. Inset in **a** is the OM image of the device with a scale bar of 50 μm.

2. Device fabrication

The device fabrication processes were illustrated in Supplementary Fig. 10. Briefly, a WSe_2 flake with a thickness of approximately 3.6 nm (Supplementary Fig. 12b) was first fabricated on a 1 cm × 1 cm SiO_2/Si substrate via mechanical exfoliation (Supplementary Fig. 10a). GDYO film was prepared as mentioned above, and its thickness was approximately 10.8 nm (Supplementary Fig. 12c). The GDYO film was patterned to a square, and then was aligned on the WSe_2 flake with the help of an optical microscope. It is worth noting that only a small part of the WSe_2 and GDYO overlap (Supplementary Fig. 10b). Other 2D materials, i.e., hBN and MoS_2, were fabricated on other substrates. A MoS_2 flake with a thickness of approximately 4.2 nm (Supplementary Fig. 12d) was transferred onto a transparent PVA film and then was aligned on top of the WSe_2/GDYO heterostructure, acting as the floating gate (Supplementary Fig. 10c). The PVA film was dissolved by immersing the sample into deionized water. With the same method, a hBN flake and a MoS_2 flake with thicknesses of 9.2 nm and 1.4 nm (Supplementary Figs. 12e,f) were stacked on top of the MoS_2 floating gate in turn (Supplementary Figs. 10d,e), serving as the blocking layer and channel, respectively. Noteworthily, the top MoS_2 channel should not align with the bottom WSe_2 layer. Finally, the source, drain and control-gate electrodes (Cr/Au 10 nm/50 nm) were deposited by laser-direct lithography, thermal evaporation and lift-off processes (Supplementary Fig. 10f).

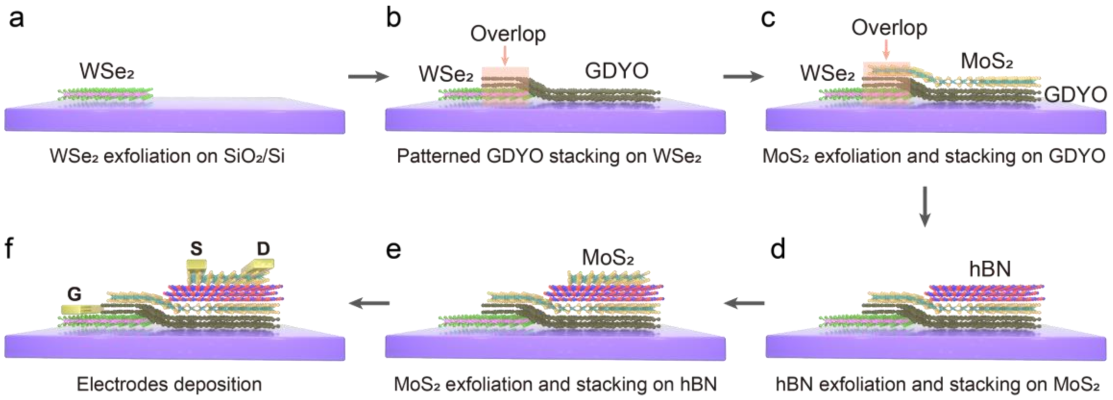

Supplementary Fig. 10| Illustration of the device fabrication processes. **a**, Mechanical exfoliation of WSe_2 flake on a SiO_2/Si substrate. **b**, Stacking the pre-patterned GDYO film on WSe_2 flake with a small overlap. **c**, Stacking the mechanically exfoliated MoS_2 flake on top of GDYO film as the floating gate. **d**, Stacking the mechanically exfoliated hBN flake on top of MoS_2/GDYO heterostructure as the blocking layer. **e**, Stacking the mechanically exfoliated MoS_2 flake on top of hBN as the channel. **f**, Deposition of the source, drain and control-gate electrodes.

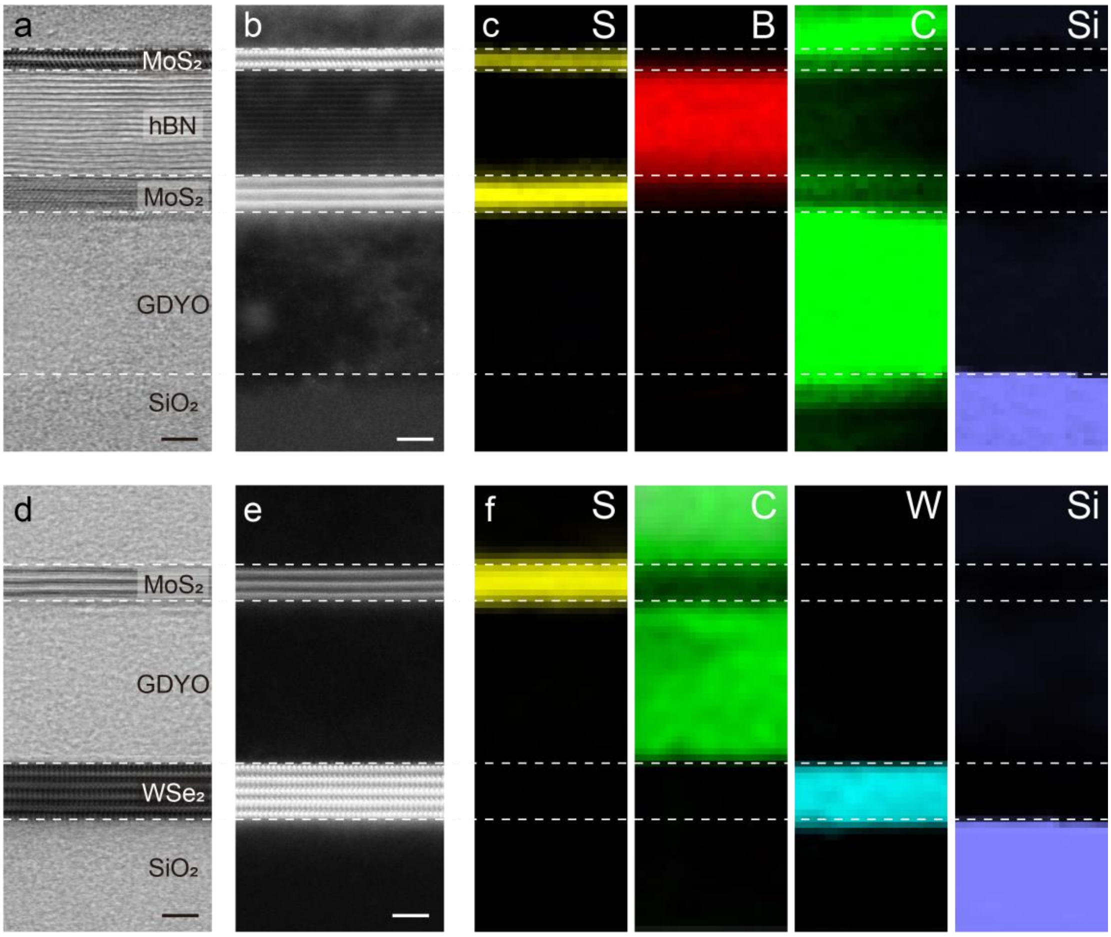

Supplementary Fig. 11| STEM and EELS characterization for MoS_2/hBN/MoS_2/GDYO and MoS_2/GDYO/WSe_2 heterostructures. **a**,**b**, Bright-filed (**a**) and HAADF (**b**) images of the MoS_2/hBN/MoS_2/GDYO heterostructure on SiO_2. **c**, EELS mapping images obtained on the cross section of the MoS_2/hBN/MoS_2/GDYO/SiO_2 structure. **d**,**e**, Bright-filed (**d**) and HAADF (**e**) images of the MoS_2/GDYO/WSe_2 heterostructure on SiO_2. **f**, EELS mapping images obtained on the cross section of the MoS_2/GDYO/WSe_2/SiO_2 structure. Scale bars, 2 nm.

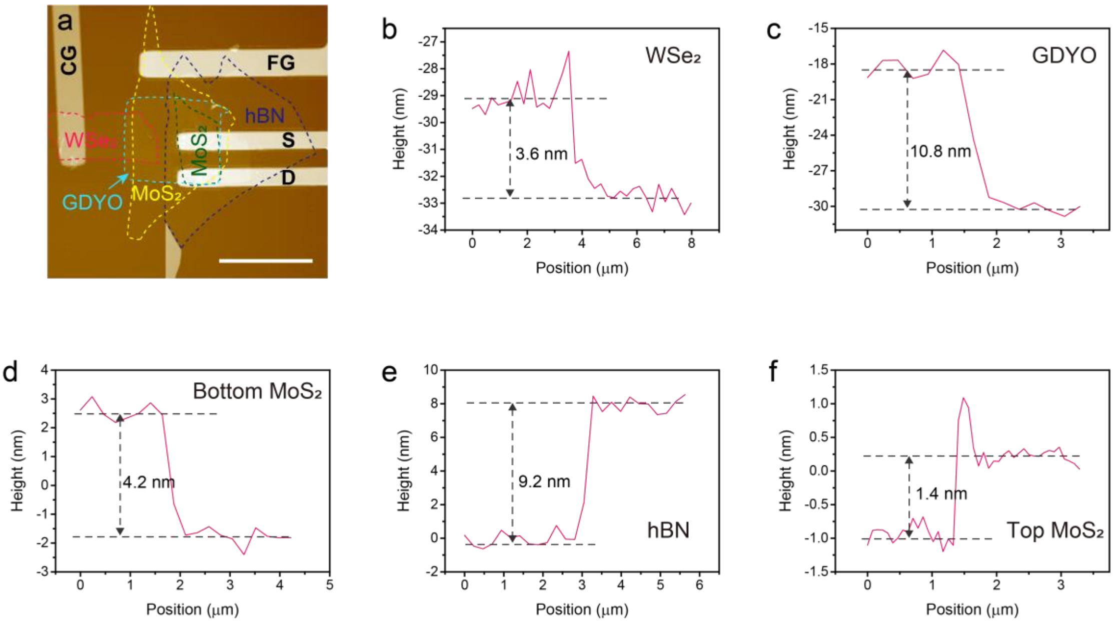

Supplementary Fig. 12| Thickness characterization of the as-fabricated memory device. **a**, AFM image of the as-fabricated memory device. The stacking order of the heterostructures is marked from bottom to top by pink, cyan, yellow, blue and green dashed lines representing the control-gate layer WSe_2, TS layer GDYO, floating-gate layer MoS_2, blocking layer hBN and channel MoS_2, respectively. Scale bar, 20 μm. **b**–**f**, The thickness of WSe_2 (**b**), GDYO (**c**), bottom MoS_2 (**d**), hBN (**e**) and top MoS_2 (**f**).

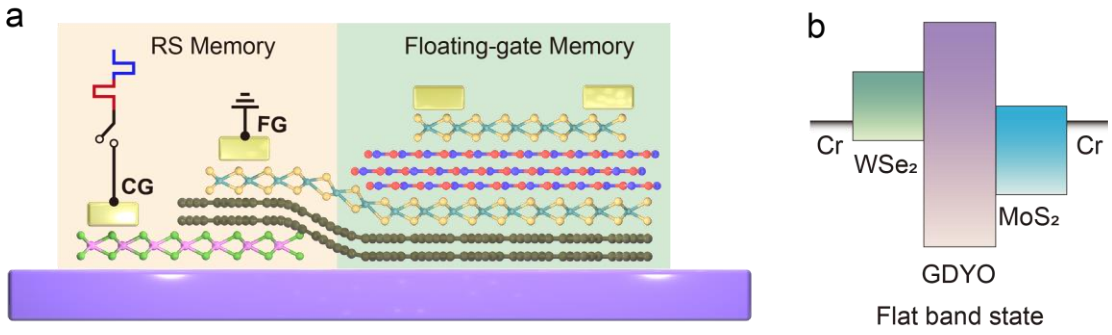

Supplementary Fig. 13| TS device based on WSe_2/GDYO/MoS_2 heterostructure. **a**, Circuit for the measurement of the switching behaviors of the TS memory. Negative and positive voltage is applied to the WSe_2 terminal and the MoS_2 terminal is grounded. **b**, The flat band diagrams of the WSe_2/GDYO/MoS_2 heterostructure.

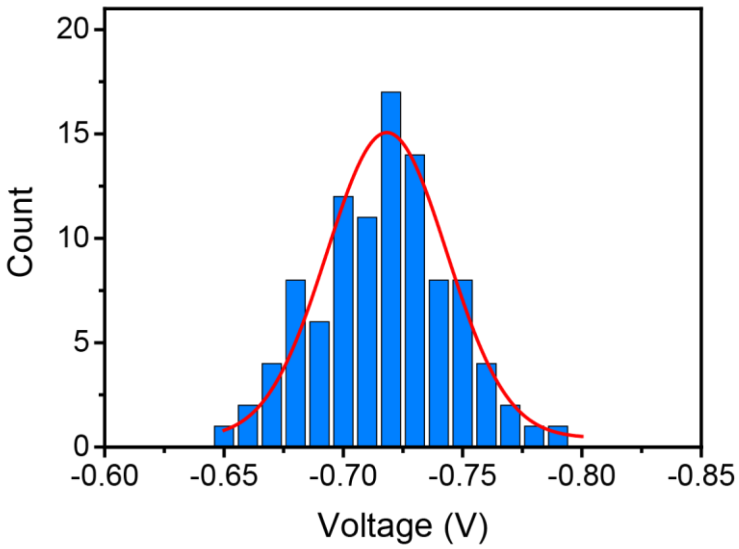

Supplementary Fig. 14| Statistical distribution of the SET voltages of the GDYO-based TS device during 100 cycles.

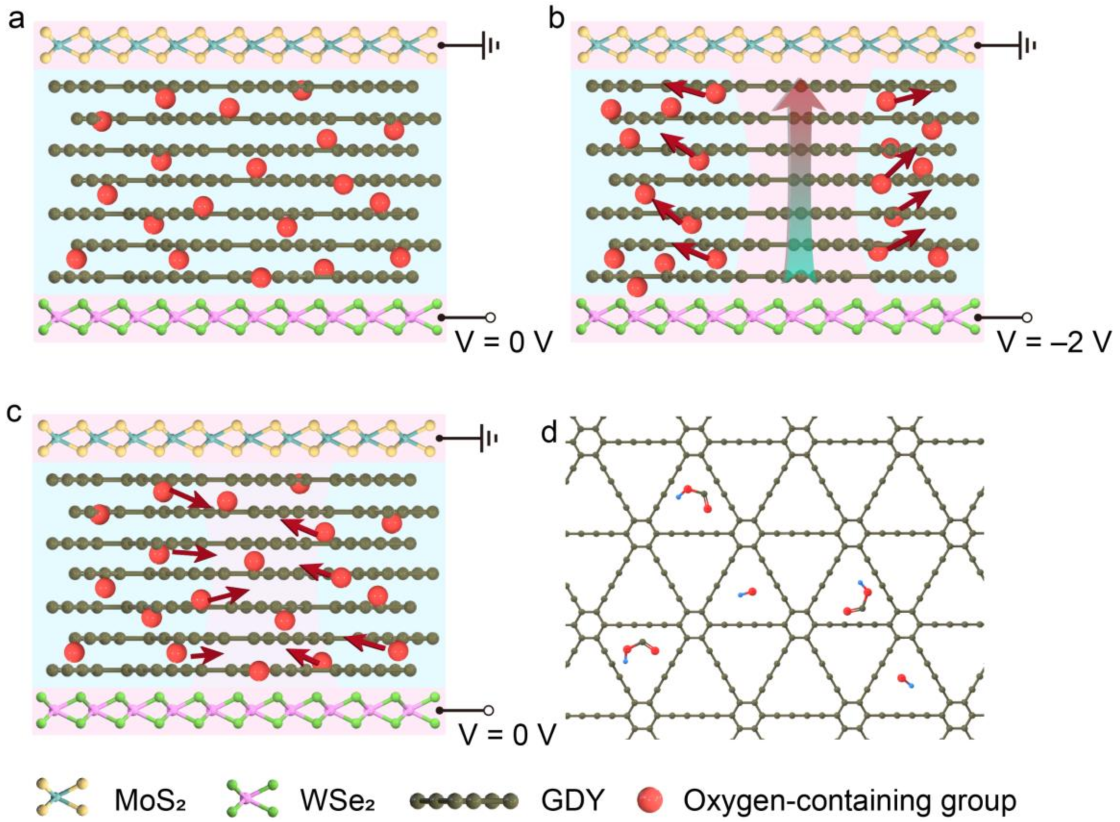

Supplementary Fig. 15| Schematic illustration of the mechanism for the device conductance switching. **a**, GDYO film containing numerous oxygen-containing groups at initial HRS. **b**, By applying a bias voltage, the oxygen-containing groups migrate, forming GDY conductive filaments in the oxygen-deficient regions. As a result, the device switches from HRS to LRS. **c**, After removing the bias voltage, the concentration difference of oxygen-containing groups drive the diffusion of oxygen-containing groups back to oxygen-deficient regions, rupturing GDY conductance filaments. Thus the device returns back to its initial HRS spontaneously. **d**, Illustration of the ordered porous structure of GDY that enables the diffusion of oxygen-containing groups through the GDY layer.

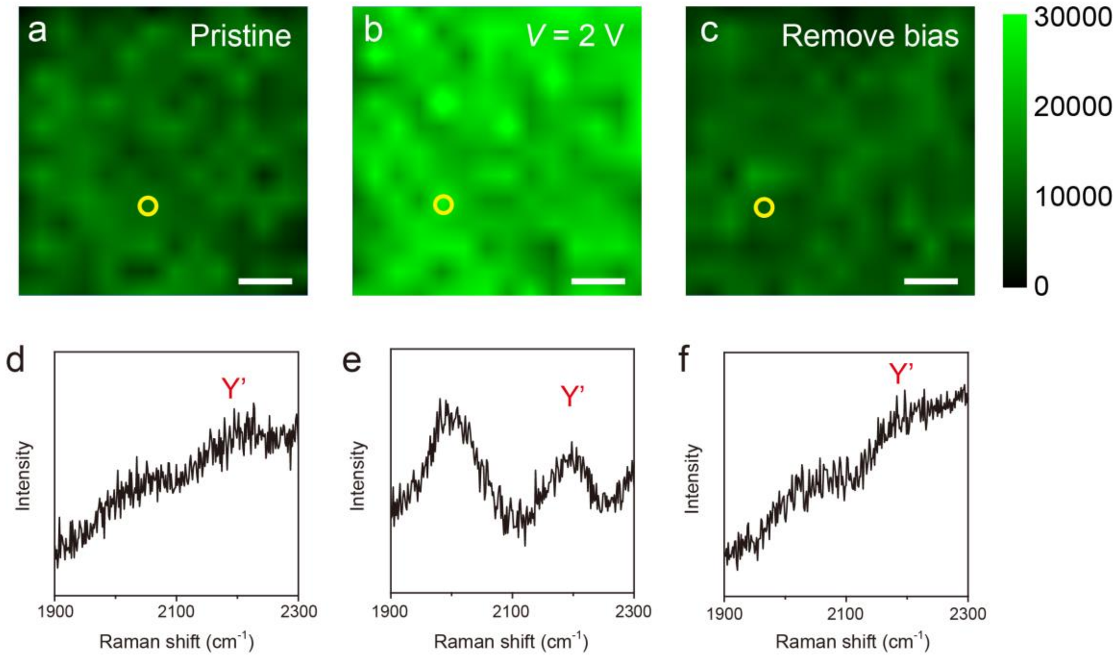

Supplementary Fig. 16| Raman Y' band maps of GDYO film with and without a bias. **a**–**c**, The Raman Y' band maps measured at pristine state (**a**), applying a 2 V bias voltage (**b**) and after removing the bias voltage (**c**). Scale bars, 2 μm. **d**–**f**, Raman profiles of the yellow point in **a**–**c**, respectively. Since the main oxidation sites of GDY are the *sp*-hybrid carbon atoms, the Raman Y' band corresponding to C≡C bond has an obvious difference between GDY and GDYO. For the pristine GDYO before applying bias voltage, its Raman Y' band is quite weak. While applying a 2 V bias voltage, the intensity of Raman Y' band has a significant enhancement, demonstrating the formation of GDY as the conductive filaments. After removing the bias voltage, the intensity of Raman Y' band returns to its initial level, which indicates that the GDY filaments are disappeared.

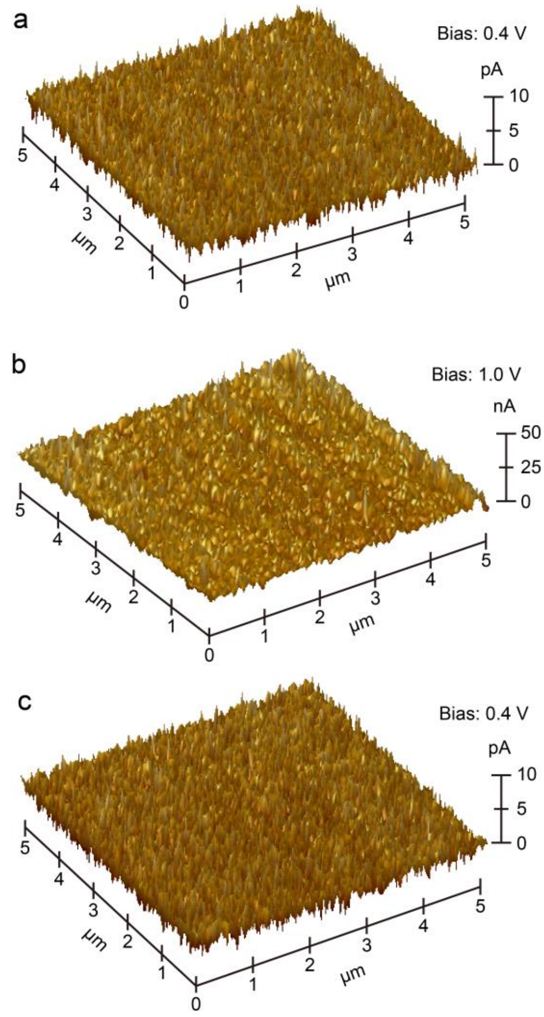

Supplementary Fig. 17| Conductive AFM images of GDYO film measured with different bias voltages. **a**, While a bias voltage (0.4 V) smaller than V_{SET} (approximately 0.7 V) was applied, the GDYO film remains at HRS with a picoampere current level. **b**, While a bias voltage (1.0 V) exceeding V_{SET} was applied, the GDYO film switched to LRS with a nanoampere current level, demonstrating the formation of GDY CFs. **c**, Followed by the measurement in **b**, a bias voltage of 0.4 V was applied again, and the GDYO film returned to its HRS with a picoampere current level, which indicates the rupture of GDY CFs after removing the bias voltage. Noteworthily, the picoampere current level in **a** and **c** is the measuring limit of the instrument.

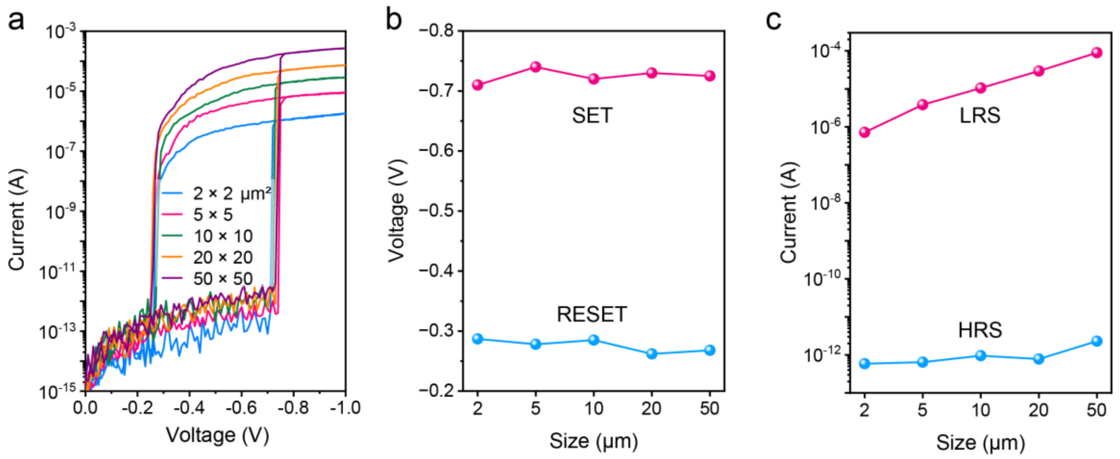

Supplementary Fig. 18| Resistive switching characteristics of the TS devices with different GDYO areas. **a**, *I*-*V* curves of the devices with GDYO areas scaling from 50 × 50 μm^2 to 2 × 2 μm^2. **b**,**c**, Distribution of the SET/RESET voltages (**b**) and HRS/LRS currents (**c**) of the devices with different sizes. These devices possess similar SET and RESET voltages due to their localized filamentary switching nature[8], while the LRS currents have a proportional increase with device size increasing.

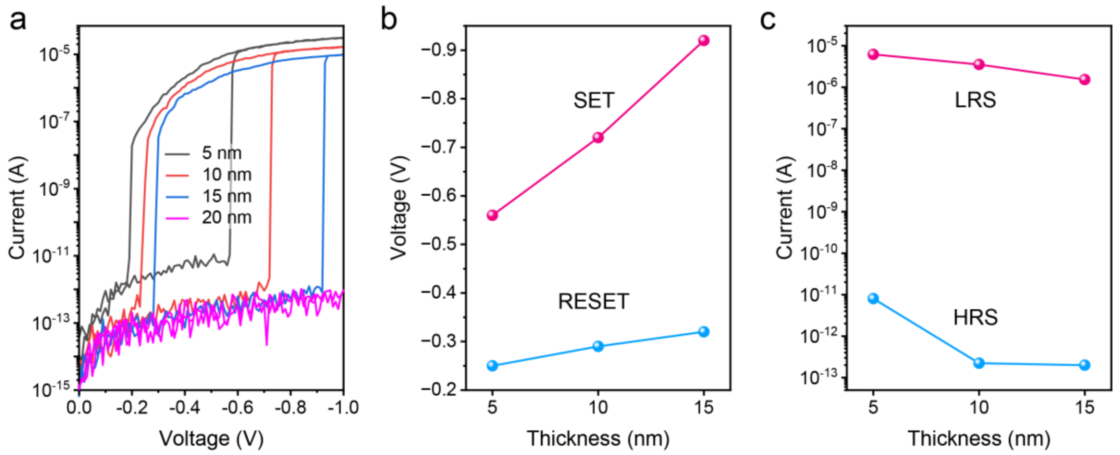

Supplementary Fig. 19| Resistive switching characteristics of the TS devices with different GDYO thickness. **a**, *I-V* curves of the devices with GDYO thickness of 5 nm, 10 nm, 15 nm and 20 nm. **b**,**c**, Distribution of the SET/RESET voltages (**b**) and HRS/LRS currents (**c**) of the devices with different thickness. With the increase of GDYO thickness, the SET/RESET voltages of the device have an obvious increase, while their LRS currents decrease correspondingly. For the device with a 20 nm thick GDYO, its SET voltage exceeds –1 V (approximately –1.3 V as shown in Supplementary Fig. 31), and thus it maintained at HRS during the voltage sweeping from 0 V to –1 V and back to 0 V. Noteworthily, for the device with a 5 nm thick GDYO, its HRS current is approximately two orders of magnitude larger than that of other devices, which might lead to the leakage of electrons from floating gate and degrade the storage performance of the memory device. Thus the optimal GDYO thickness evaluating by the SET voltage and HRS current is ~10 nm for the memory device as demonstrated in Supplementary Figs. 29 and 30.

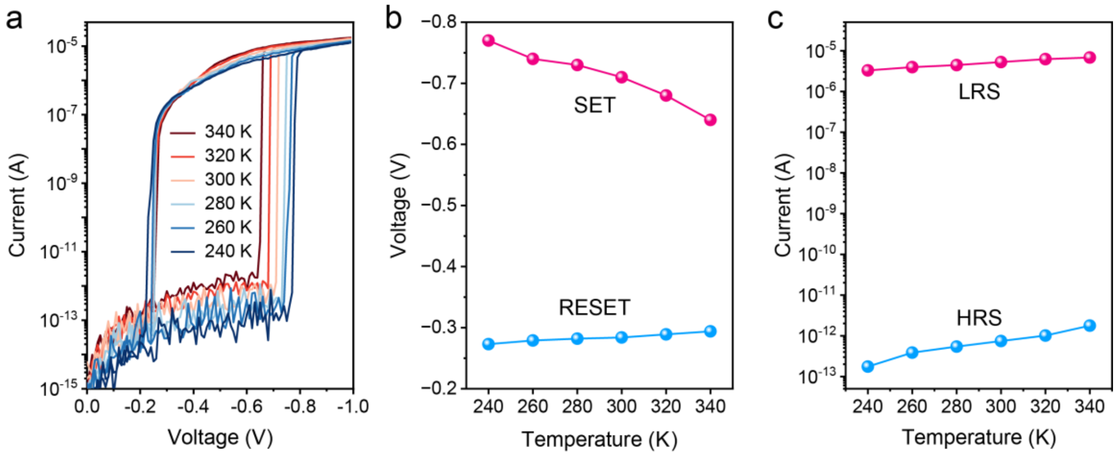

Supplementary Fig. 20| Temperature-dependent resistive switching characteristics of the TS device. **a**, *I-V* curves of the devices measured at temperature ranging from 240 K to 340 K. **b**,**c**, Distribution of the SET/RESET voltages (**b**) and HRS/LRS currents (**c**) of the devices as a function of temperature. An obviously decreased SET voltage was observed with temperature increasing, since higher temperature can intensify the movement of oxygen-containing groups and accelerate the formation of GDY CFs. As well, higher temperature leads to a larger HRS current, which would sacrifice the retention performance of the memory device as demonstrated in Supplementary Fig. 44.

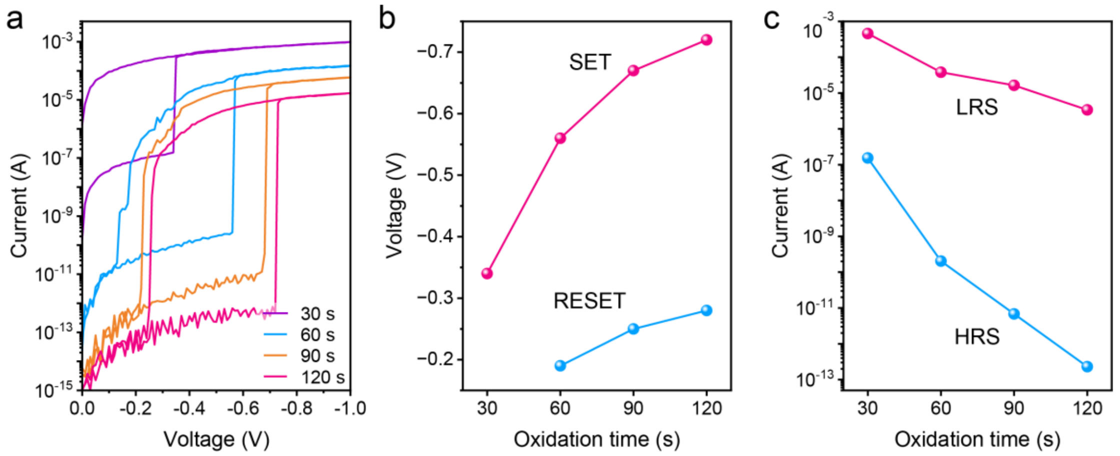

Supplementary Fig. 21| Resistive switching characteristics of the TS devices with different oxidation treatment time. **a**, *I-V* curves of the devices with oxidation time ranging from 30 s to 120 s. **b**,**c**, Distribution of the SET/RESET voltages (**b**) and HRS/LRS currents (**c**) of the devices as a function of oxidation time. The oxygen content in GDYO increases while prolonging UV-ozone treatment time. With the decrease of oxidation time (oxygen content), the on/off ratio of the device has a serious degradation due to the significant increase of the HRS current, and the SET/RESET voltages also show an obvious decrease. Noteworthily, for the device with a 30 s oxidation time, it cannot switch back to HRS after voltage sweeping due to the low concentration of oxygen-containing groups in this device.

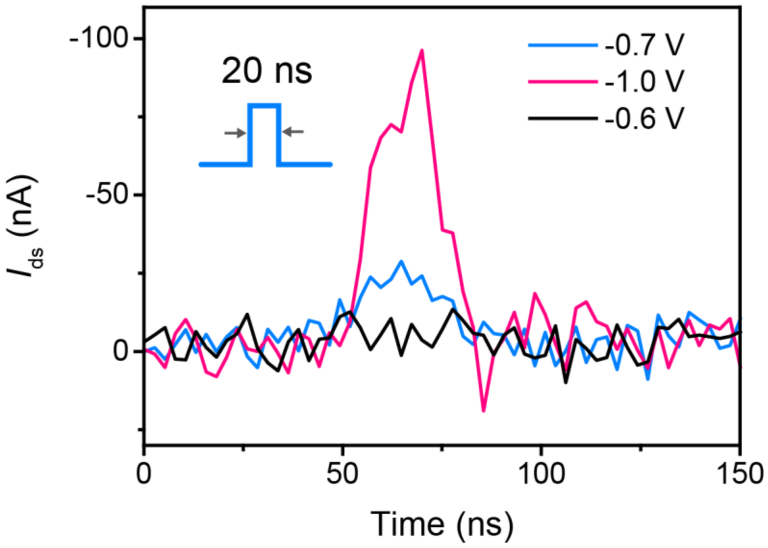

Supplementary Fig. 22| Transient response of the GDYO-based TS device triggered by a 20 ns V_{SET} pulse with amplitude of –1.0 V (red), –0.7 V (blue) and –0.6 V (black), respectively. The –0.6 V voltage pulse cannot switch the device.

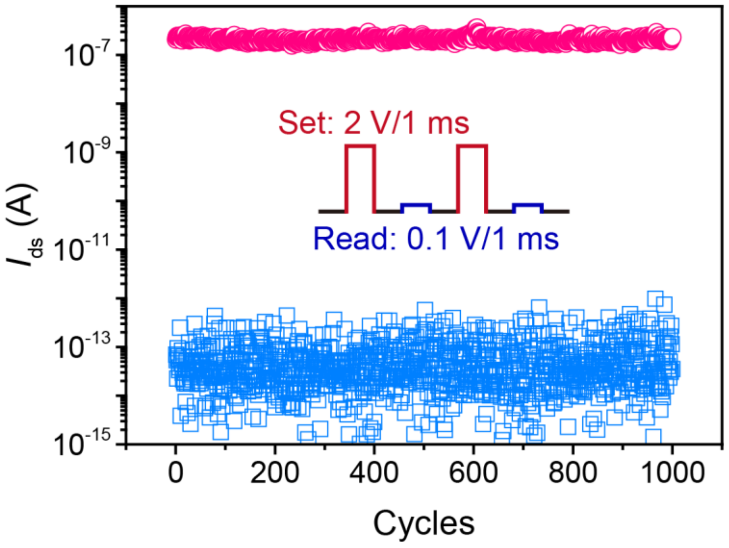

Supplementary Fig. 23| Endurance test of the GDYO-based TS device by applying periodic voltage pulses which consist of a 2 V/1 ms set pulse followed by a 0.1 V/1 ms read pulse. The interval between the set and read pulses is 1 ms.

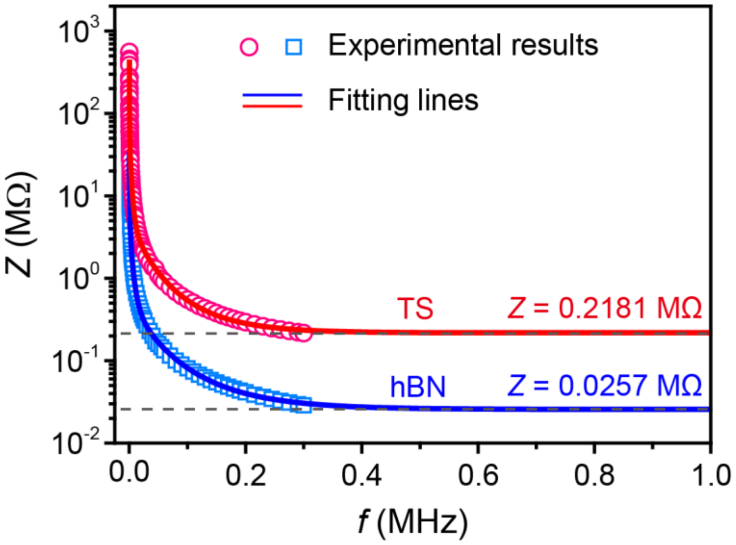

Supplementary Fig. 24| Impedance of the GDYO-based TS device and hBN as a function of frequency. To estimate the effective voltage applied to the GDYO-based TS device for the SET operation, we first measured the impedance of the GDYO-based TS device and hBN. Limited by the instrument, here the frequency can only be measured to 0.3 MHz. Nevertheless, our results can be well fitted by a three-exponential equation, and the fitting results indicate that the impedance of the TS and hBN at high frequency ($f > 0.5$ MHz) are 0.2181 MΩ and 0.0257 MΩ, respectively. Since a 20 ns operation speed corresponds to an ultrahigh frequency of 50 MHz, the impedance of the GDYO-based TS device is almost one order of magnitude larger than that of hBN. As a result, most of the applied gate voltage (V_{CG}) would be loaded on the TS device.

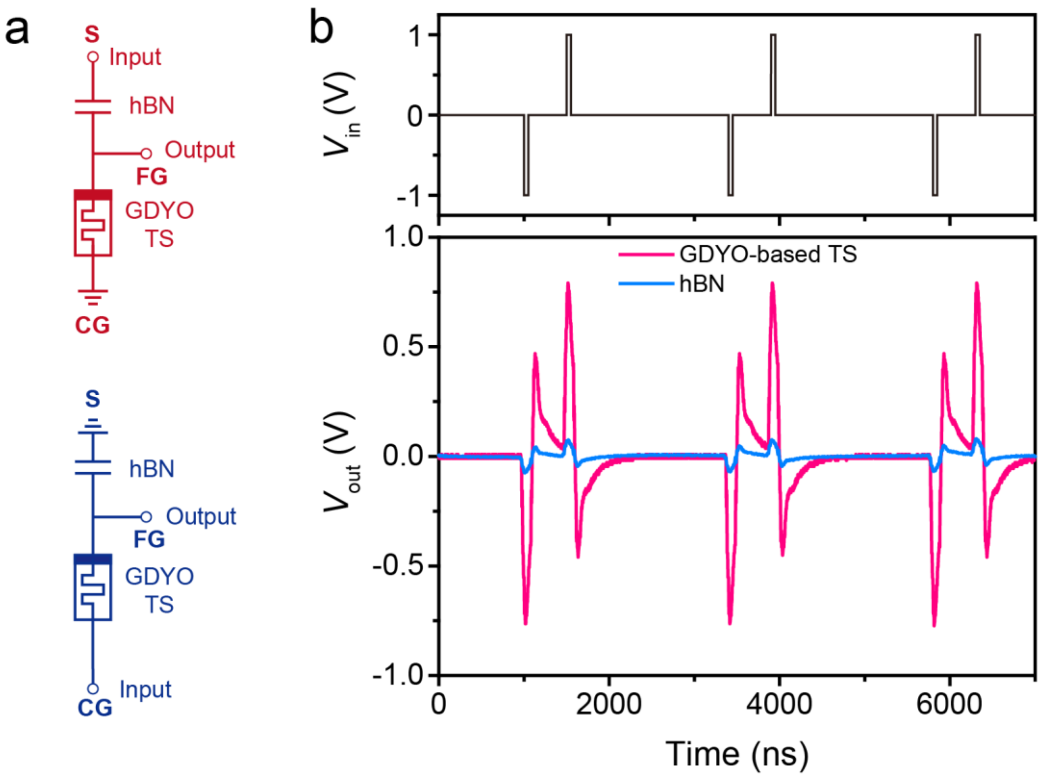

Supplementary Fig. 25| Effective voltage loaded on the GDYO-based TS device and hBN. **a**, Circuits used for the measurement of the voltage across the GDYO-based TS device (red) and hBN (blue), respectively. **b**, The output voltages measured using the circuits as illustrated in **a**. The input periodic waveform consists of ±1 V/50 ns pulsed voltages with an interval of 500 ns. The results demonstrate that the output voltage across the GDYO-based TS device is approximately ±0.8 V, whereas the output voltage across the hBN is only ±0.08 V. Thus we can conclude that approximately 80% of the applied V_{CG} voltage can be effectively loaded on the GDYO-based TS device.

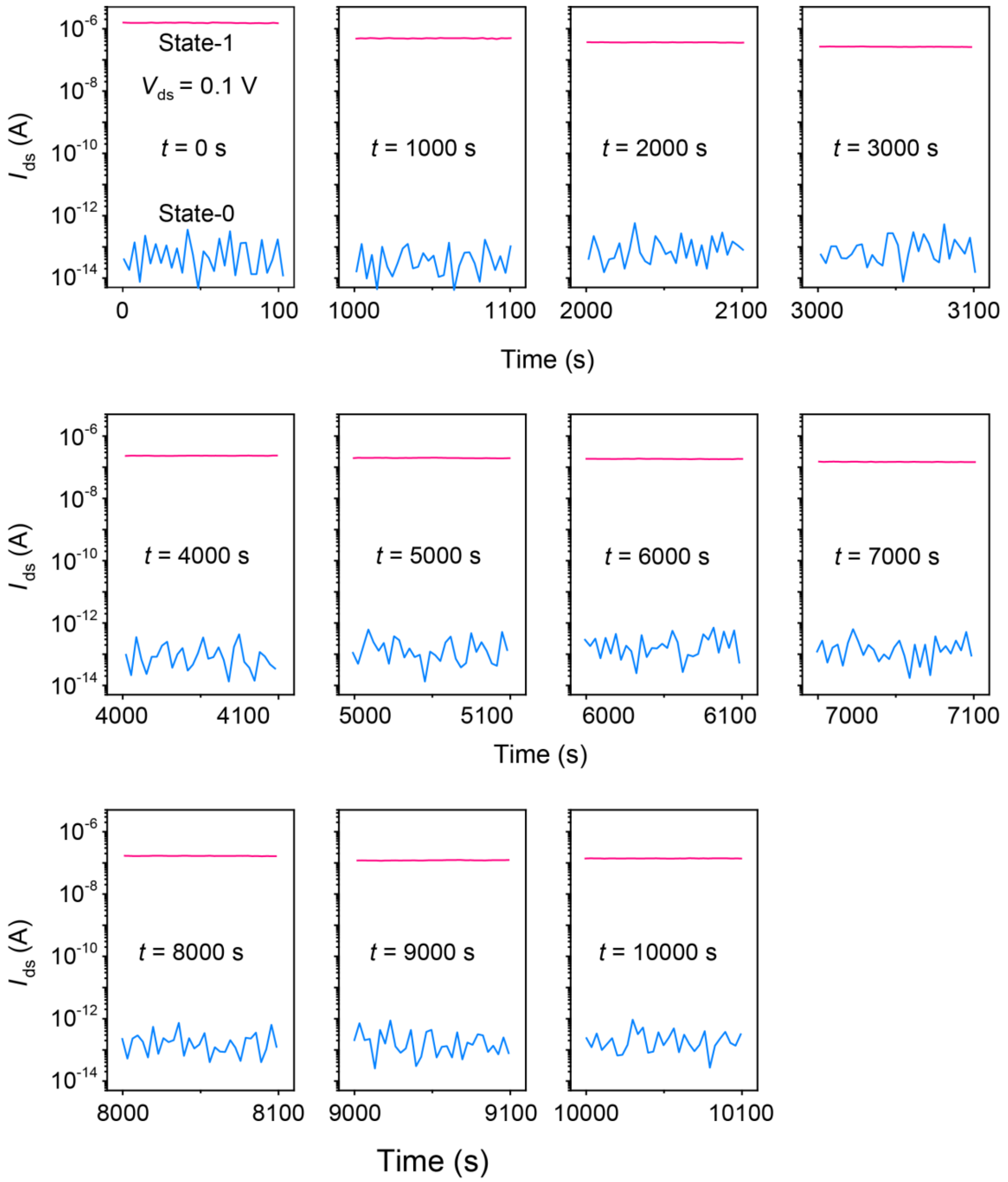

Supplementary Fig. 26| The readout currents of the memory device after the writing (pink) and erasing (blue) operations with a fixed interval of 1000 s. The readout operations were performed with a fixed V_{ds} of 0.1 V at V_{CG} = 0 V.

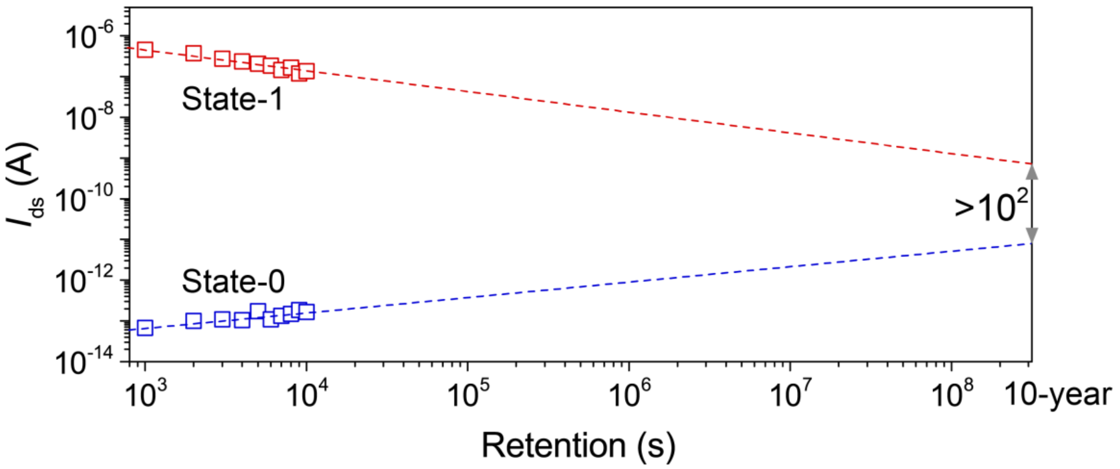

Supplementary Fig. 27| 10 years linear extrapolation of the state-1/state-0 currents. The state-1/stato-0 ratio still exceeds 10^2 when the retention curves are extrapolated to 10-year, demonstrating an ultralong retention time of our memory device.

Supplementary Table 1| Parameters of the nonvolatile based on 2D van der Waals heterostructures.[a)]

Structure	Mechanism	Write		Erase		Retention	Endurance	On/Off Ratio	Multilevel Storage	Ref.
		Voltage	Speed	Voltage	Speed					
MoS_2/hBN/MoS_2/ GDYO/WSe_2	Direct charge injection	–2 V	20 ns	2 V	20 ns	10-year	1000	10^7	8 levels	This work
InSe/hBN/MLG	FN tunnelling	20 V	21 ns	–20 V	21 ns	10-year	2000	10^{10}	16 levels	[9]
MoS_2/hBN/MLG	FN tunnelling	30 V	20 ns	–30 V	20 ns	10-year	1390	10^6	–	[10]
MoS_2/hBN/GDY/Gr	Direct charge injection	30 mV	8 ns	–0.5 V	1 µs	10^4 s	1000	10^6	6 levels	[11]
WSe_2/MoS_2-hBN/HfS_2	Direct charge injection	–1.8 V	15 ns	5 V	1 ms	10 s	100	10^3	–	[12]
WSe_2/hBN/MoS_2-WSe_2	Direct charge injection	5.5 V	40 ns	–5.5 V	40 ns	14 s	100	10^3	–	[13]
WSe_2/MoS_2-hBN/HfS_2/HZO	Direct injection/ FeFET	–4.2 V	40 ns	5 V	10 ms	63.5 s	–	10^3	–	[14]
MoS_2/hBN/Gr	FN tunnelling	–6 V	5 ms	6 V	5 ms	10^4 s	10^5	10^9	–	[15]
Gr/hBN/MoS_2	FN tunnelling	15 V	100 µs	–15 V	100 µs	1400 s	100	10^3	–	[16]

MoS_2/cPVP/Au NPs	FN tunnelling	–20 V	1 s	100 V	1 s	10^5 s	250	10^3	8 levels	17
MoS_2/hBN/Gr	FN tunnelling	60 V	1 s	–60 V	1 s	10^4 s	500	10^6	7 levels	18
ReS_2/hBN/Gr	FN tunnelling	50 V	100 ms	–80 V	100 ms	2000 s	15	10^5	8 levels	19
MoS_2/HfO_2/Gr	FN tunnelling	18 V	100 ms	–18 V	100 ms	2000 s	120	10^4	–	20
MoS_2/Al_2O_3/HfO_2/Al_2O_3	FN tunnelling	26 V	3 s	–26 V	3 s	2000 s	120	10^4	–	21
WSe_2/Al_2O_3/CdSe QDs	FN tunnelling	18 V	1 s	–18 V	8 s	500 s	400	10^4	4 levels	22
WSe_2/Al_2O_3/HfO_2/Al_2O_3	FN tunnelling	40 V	100 ms	–40 V	100 ms	2000 s	700	100	–	23
ReS_2/hBN/Gr	FN tunnelling	20 V	1 μs	–20 V	1 μs	4000 s	400	10^8	4 levels	24
MoS_2/hBN/MoS_2	FN tunnelling	40 V	1 ms	–40 V	1 ms	2×10^4 s	100	10^6	–	25
BP/Al_2O_3	Interface charge trap	20 V	100 ms	–20 V	100 ms	1000 s	100	5×10^4	4 levels	26
MoS_2/Al_2O_3/ZnO	Interface charge trap	10 V	100 μs	–10 V	100 μs	10^4 s	10^3	10^4	–	27
WSe_2/DDQ	Interface charge trap	100 V	5 ms	–100 V	5 ms	1000 s	400	10^3	–	28

WSe_2/hBN	Charge trap	–80 V	7 s	0.5 W/cm^2	5 s	10-year	200	10^6	128 levels	29
MoS_2/GDY	Charge trap	–80 V	1 s	80 V	8 s	10^4	3000	8×10^7	9 levels	30
MoS_2/NBIT FeFET	FeFET	2 V	10 ms	–3 V	10 ms	720 s	100	10^4	–	31
MoS_2/hBN/CIPS	FeFET	60 V	2 s	–60 V	2 s	10^4 s	300	10^7	–	32

[a)] For some optoelectronic memory devices that can operate in both electrical and optical pathways, parameters for electrical operations are summarized.

Notes: HZO: $HfZrO_4$; NBIT: $Na_{0.5}Bi_{4.5}Ti_4O_{15}$; FeFET: ferroelectric field-effect transistor; CIPS: $CuInP_2S_6$; DDQ: 2,3-dichloro-5,6-dicyano-1,4-benzoquinone; cPVP: crosslinked poly(4-vinylphenol); QDs: quantum dots; NPs: nanoparticles; BP: black phosphorus.

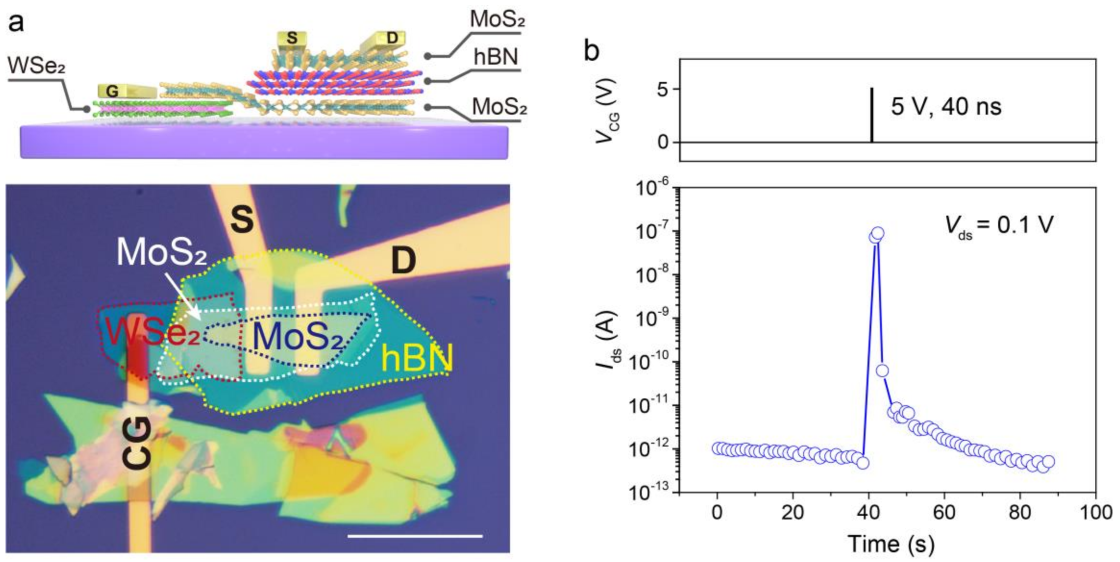

Supplementary Fig. 28| Memory device with a structure of MoS_2/hBN/MoS_2/WSe_2. **a**, Illustration of the memory device and its OM image. The boundaries of WSe_2, MoS_2 (bottom), hBN and MoS_2 (top) are marked by red, white, yellow and blue dashed lines, respectively. Scale bar, 30 µm. **b**, The response of the memory device triggered by a 40 ns V_{CG} pulse. While applying a positive V_{CG} pulse, holes are directly injected into the floating-gate MoS_2 layer through the WSe_2/MoS_2 p-n junction. The injected holes in MoS_2 would induce the threshold voltage of the top MoS_2 channel to shift left, and thus the device is switched to on-state. However, the relaxation of charges through the WSe_2/MoS_2 junction is unavoidable due to the thermal diffusion in the p-n junction area, resulting in temporary storage performance (~10 s).

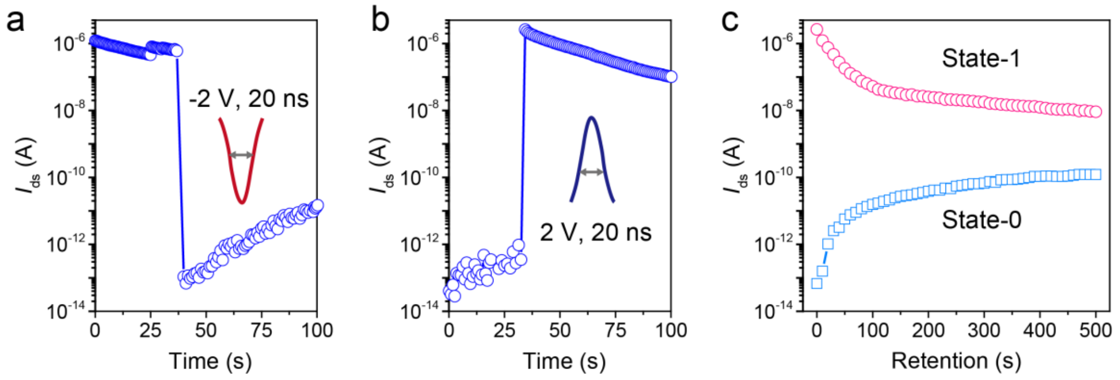

Supplementary Fig. 29| Storage performance of a memory device with a thin GDYO layer (3 nm). **a**,**b**, The successful ultrafast writing (**a**) and erasing (**b**) operations of the device by applying a 20 ns V_{CG} pulses with amplitude of ±2 V, respectively. **c**, the retention performance of the device with a 3 nm GDYO layer. V_{ds} is fixed as 0.1 V for the readout operation.

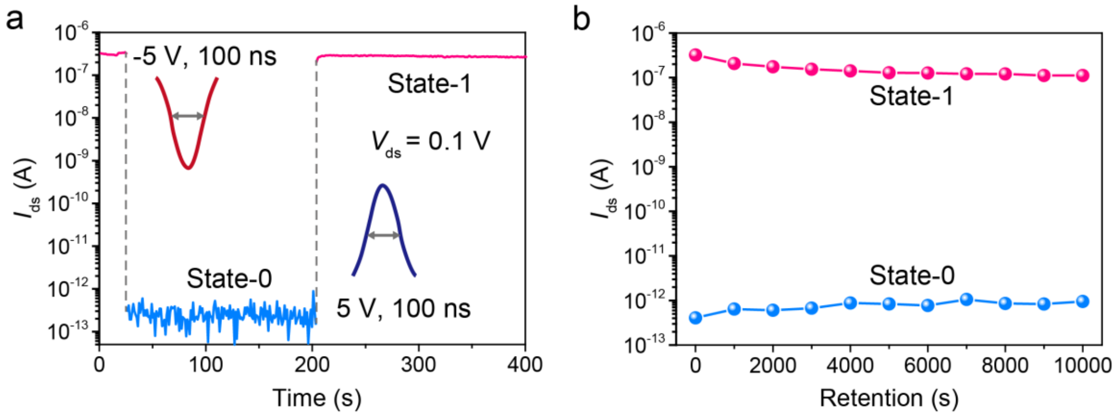

Supplementary Fig. 30| Storage performance of a memory device with a thick GDYO layer (20 nm). **a**, The switching of the channel currents by applying –5 V/+5 V V_{CG} pulses with FWHM of 100 ns for the writing and erasing operations, respectively. **b**, the retention performance of the device with a 20 nm GDYO layer. V_{ds} is fixed as 0.1 V for the readout operation.

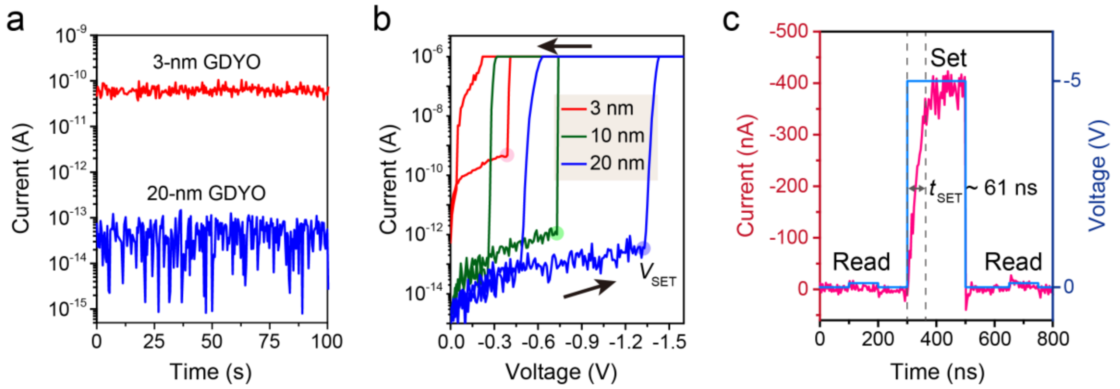

Supplementary Fig. 31| GDYO-based TS memories with different thicknesses of GDYO layer. **a**, The off-state currents of the devices with GDYO thickness of 3 nm (red) and 20 nm (blue), respectively. A fixed V_{ds} of 0.1 V was used to read the channel current. **b**, TS characteristics of devices with GDYO thickness of 3 nm (red), 10 nm (green) and 20 nm (blue), respectively. The red, green and blue circles mark the V_{SET} of these devices. **c**, Switching time of the TS device with a 20 nm GDYO layer. A –5 V/200 ns voltage pulse was used to set the device, and the read pulses were –0.1 V/100 ns. The off-state conductance of the device with a 3 nm GDYO layer is several orders of magnitude larger than that of the device with a 20 nm GDYO layer. For the memory device with a 3 nm GDYO layer, the GDYO TS layer cannot be completely turned off due to its large off-state conductance, leading to the escape of the injected electrons and thus poor retention characteristics as shown in Supplementary Fig. 29. On the other hand, with the increase of GDYO thickness, an increased SET voltage and longer switching time were observed, and thus V_{CG} pulses with larger amplitude and longer duration are required to switch the GDYO TS layer for the writing/erasing operations (Supplementary Fig. 30).

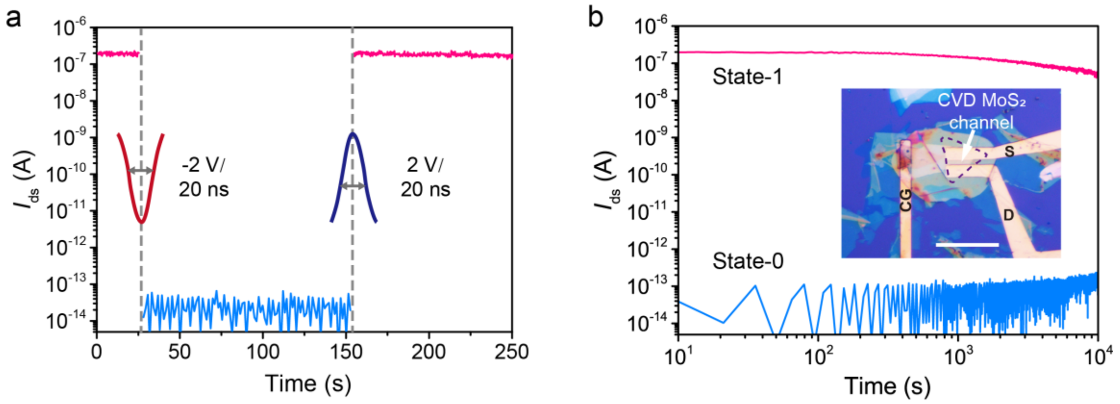

Supplementary Fig. 32| Storage performance of a memory device using a CVD-grown monolayer MoS_2 as the channel. **a**, The switching of the channel currents by applying ±2 V/20 ns V_{CG} pulses for the writing and erasing operations, respectively. **b**, the retention characteristics of the device at State-1 and State-0. V_{ds} is fixed as 0.1 V for the readout operation. Inset is the OM image of the device, and the CVD-grown MoS_2 is marked by the purple dotted triangle. Scale bar, 30 μm.

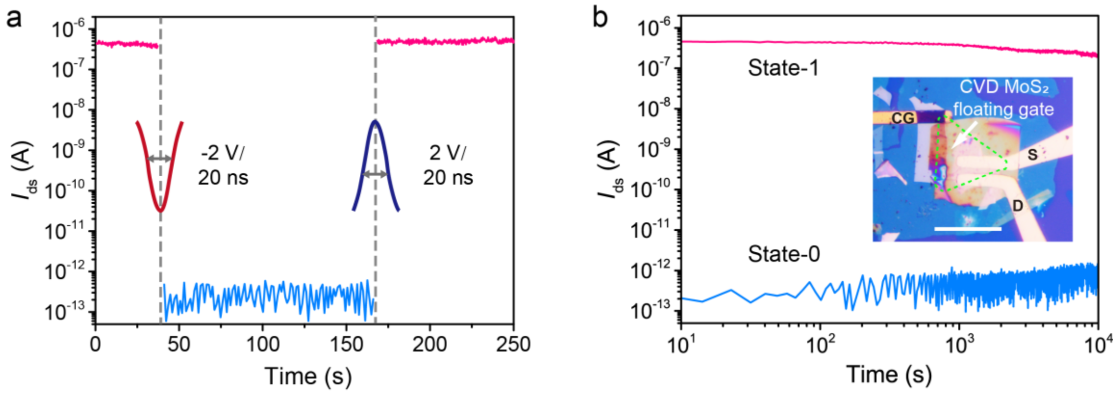

Supplementary Fig. 33| Storage performance of a memory device using a CVD-grown monolayer MoS_2 as the floating gate. **a**, The switching of the channel currents by applying ±2 V/20 ns V_{CG} pulses for the writing and erasing operations, respectively. **b**, the retention characteristics of the device at State-1 and State-0. V_{ds} is fixed as 0.1 V for the readout operation. Inset is the OM image of the device, and the CVD-grown MoS_2 is marked by the green dotted triangle. Scale bar, 30 μm.

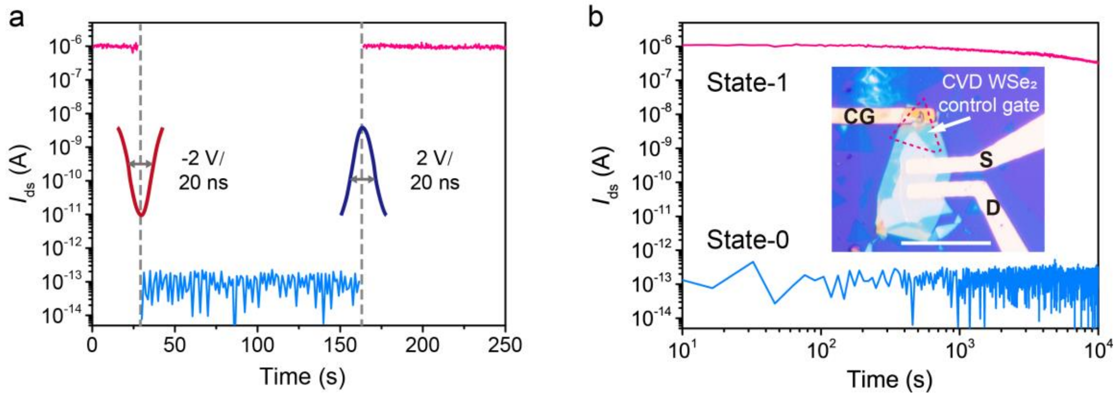

Supplementary Fig. 34| Storage performance of a memory device using a CVD-grown monolayer WSe_2 as the control gate. **a**, The switching of the channel currents by applying ±2 V/20 ns V_{CG} pulses for the writing and erasing operations, respectively. **b**, the retention characteristics of the device at State-1 and State-0. V_{ds} is fixed as 0.1 V for the readout operation. Inset is the OM image of the device, and the CVD-grown WSe_2 is marked by the red dotted triangle. Scale bar, 30 μm.

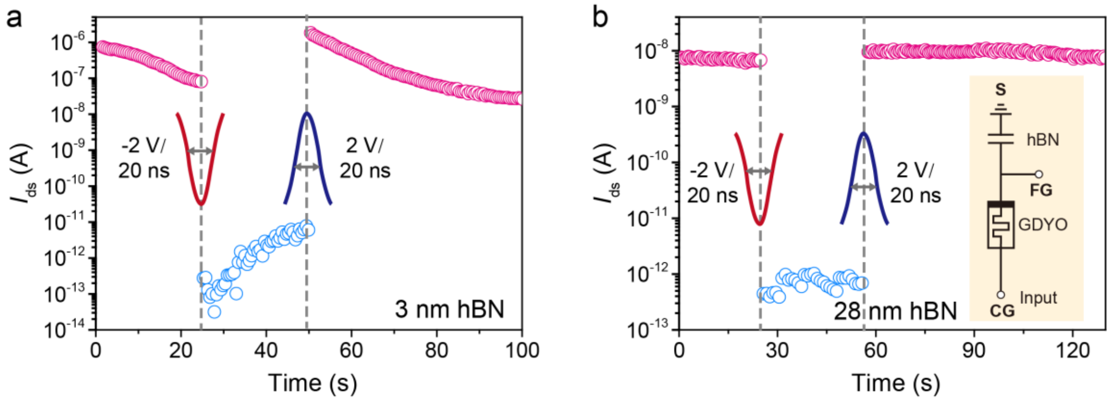

Supplementary Fig. 35| Storage performance of devices with different hBN thickness. The hBN blocking layer used in **a** and **b** were 3 nm (**a**) and 28 nm (**b**), respectively. The thickness of hBN blocking layer have a significant influence on device performance. For the device with a 3 nm hBN film, the blocking layer is too thin to restrict the charges in the floating gate, leading to the leakage of charges from floating gate to channel. Thus the device exhibits poor retention characteristics after the writing and erasing operations. The inset in **b** illustrates the equivalent circuit of the memory device while applying a V_{CG} pulse, in which the hBN and GDYO are connected in series. Considering that a thicker hBN film has a larger resistance, which will share more voltage. For the device with a 28 nm hBN layer, the effective voltage loaded on the GDYO TS layer is reduced in comparison with that of the device with a 10 nm hBN film, which affects the writing and erasing operations with a degraded on/off ratio (10^4). Thus the thickness of hBN blocking layer should also be carefully chosen, and a 10 nm thick hBN film is suitable for our memory device.

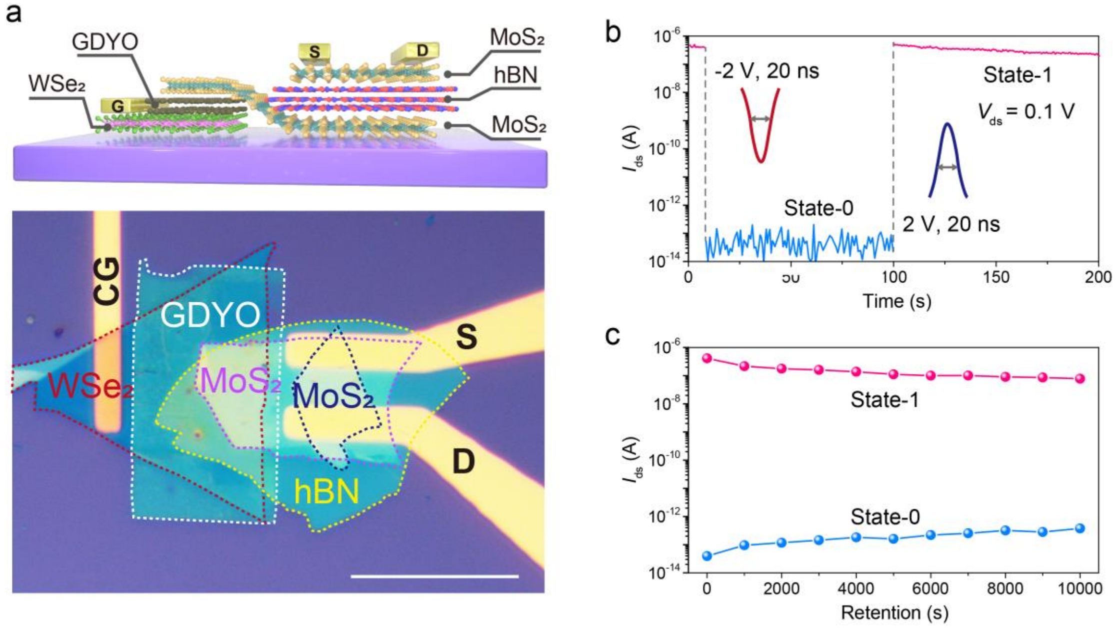

Supplementary Fig. 36| Memory device with a structure of MoS_2/hBN/MoS_2/GDYO/WSe_2 without overlapping between the top MoS_2 channel and the GDYO layer. **a**, Illustration and corresponding OM image of the device. This device was fabricated by carefully eliminating overlapping between the top MoS_2 channel and the GDYO layer. The boundaries of WSe_2, GDYO, MoS_2 (bottom), hBN and MoS_2 (top) are marked by red, white, purple, yellow and blue dashed lines, respectively. Scale bar, 30 μm. **b**, The successful writing/erasing operations of the memory device by applying a 20 ns V_{CG} voltage with amplitude of –2 V and +2 V, respectively. **c**, The long retention characteristics of the memory device at both state-0 and state-1. V_{ds} is fixed as 0.1 V for the readout operation. In this device, since the control gate and the GDYO layer are not overlapped with the top MoS_2 channel, both the electric fields induced by the gate voltage or the trapped charges in the GDYO layer cannot direct modulate the channel conductance. Thus we can conclude that the ultrafast writing/erasing operations and long retention characteristics of the memory device are attributed to the accumulated charges in the floating gate.

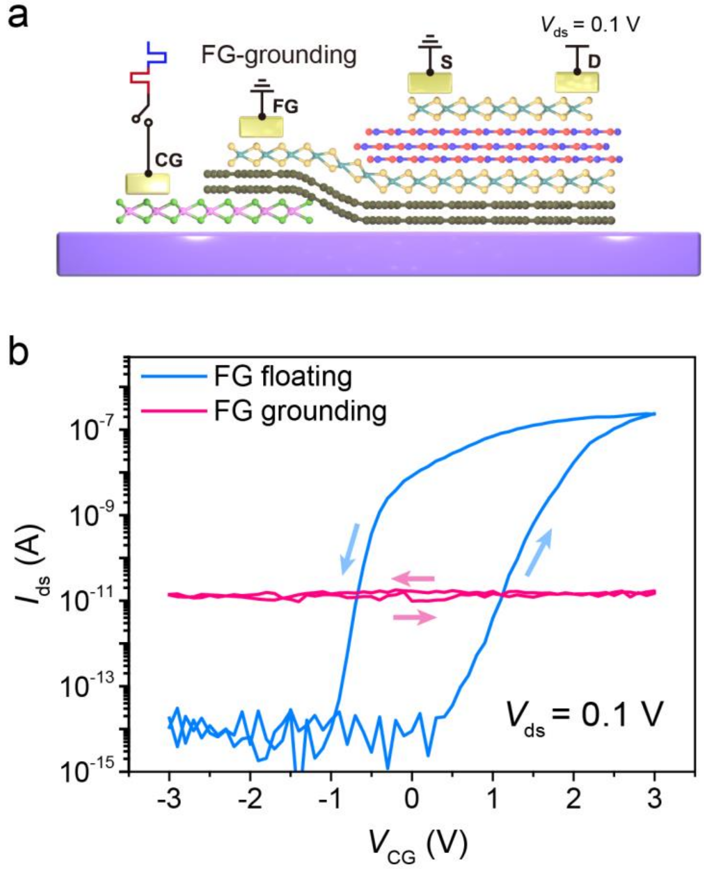

Supplementary Fig. 37| The transfer characteristics of the memory device with floating gate grounding. **a**, Illustration of the measuring circuit with FG grounding. **b**, The transfer curves of the device with FG grounding (pink) and floating (blue), respectively. The gate voltage sweeping from –3 V to 3 V and then back to –3 V was applied to the control gate and the V_{ds} was fixed as 0.1 V. Since the control gate (WSe_2) and the top MoS_2 channel are not overlapped, the channel conductance cannot be directly modulated by the control gate, but can be modulated by injected charges in the floating gate or the GDYO layer. For the case of the FG floating, the transfer curve features a counterclockwise hysteresis with the gate voltage sweeping, due to the injection and extraction of electrons from the control gate to the floating gate. On the contrary, the channel current cannot be modulated by the gate voltage while the FG is grounded. This is because the injected charges in the floating gate are guided to the ground, and no charges accumulate in the floating gate to modulate the channel conductance. Thus we can conclude that the injected charges are mainly accumulated in the floating gate rather than the GDYO layer.

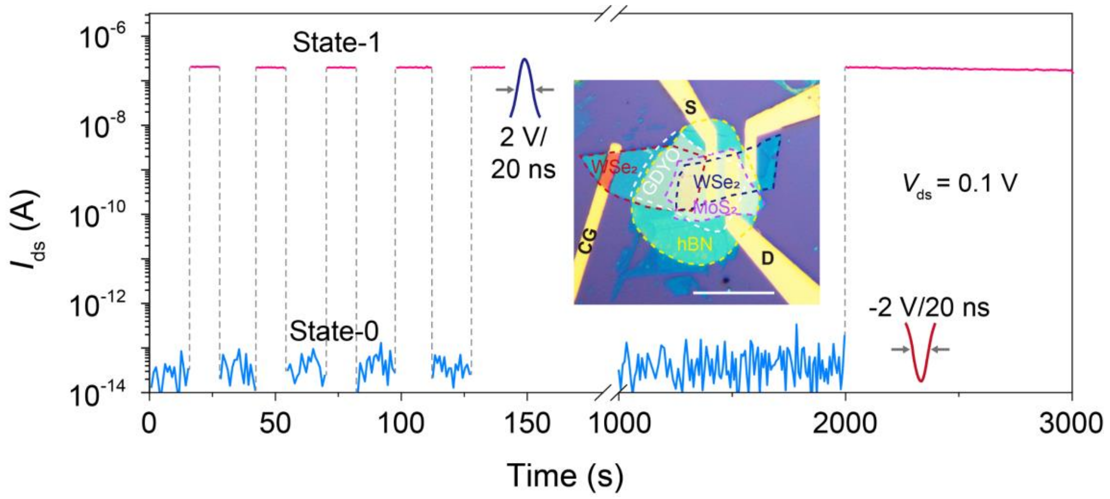

Supplementary Fig. 38| Low-voltage ultrafast nonvolatile memory using a p-type WSe_2 as the channel layer. The ultrafast writing/erasing operations can be successfully and repeatedly achieved by applying 20 ns V_{CG} pulses with amplitude of –2 V and +2 V, respectively. Noteworthily, due to p-type feature of the WSe_2 channel, the writing operation switch the device from off-state to on-state, and vice versa, which is opposite to that of the memory device based on n-type MoS_2 channel. Complementary writing and erasing operations achieved by different channel materials can greatly enrich the diversity of device performance for practical applications. The inset shows the OM image of the device, and the boundaries of WSe_2 (bottom), GDYO, MoS_2, hBN and WSe_2 (top) are marked by red, white, purple, yellow and blue dashed lines, respectively. Scale bar, 30 μm.

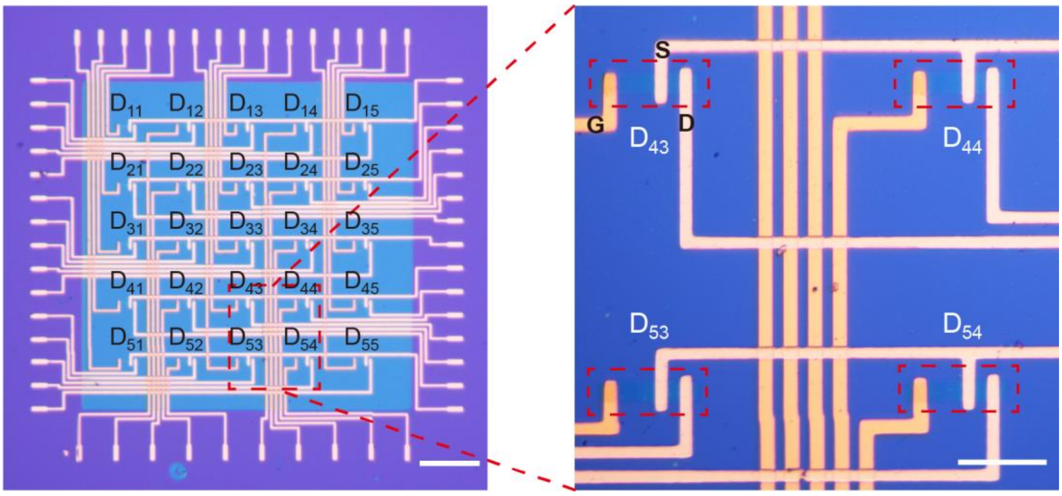

Supplementary Fig. 39| 5 × 5 device array fabricated by CVD-grown large-area MoS_2 and WSe_2 films. Scale bars, 50 μm (left) and 10 μm (right). Monolayer CVD-grown WSe_2 was first patterned into rectangle array as the control gate of the devices, followed by the deposition of Cr/Au as the gate electrodes connecting the WSe_2. Then the prepatterned GDYO and MoS_2 (CVD-grown) layers were stacked on top in turn, acting as the TS layer and floating gate, respectively. Noteworthily, a 20 nm thick HfO_2 layer instead of hBN film fabricated via atomic layer deposition (ALD) was used as the blocking layer between the floating gate and channel. Finally, prepatterned CVD-grown MoS_2 was stacked on top of the HfO_2, acting as the channel, and the source and drain electrodes (Cr/Au) were deposited.

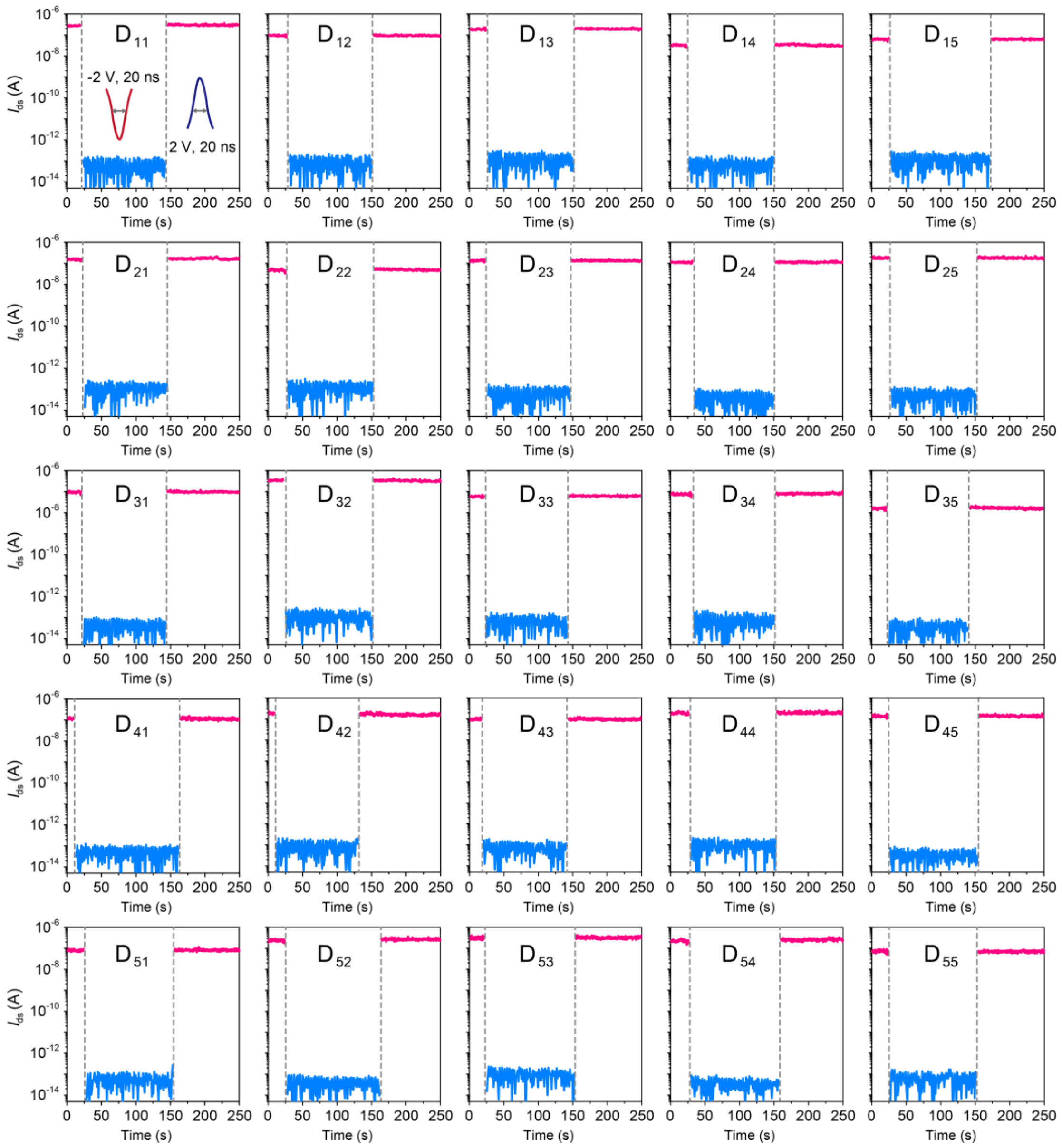

Supplementary Fig. 40| Ultrafast writing and erasing operations of 25 memory devices based on CVD-grown MoS_2 and WSe_2 as illustrated in Supplementary Fig. 38. A –2 V, 20 ns and a +2 V, 20 ns V_{CG} pulses were used for the writing and erasing operations, respectively, and the readout voltage was fixed at 0.1 V.

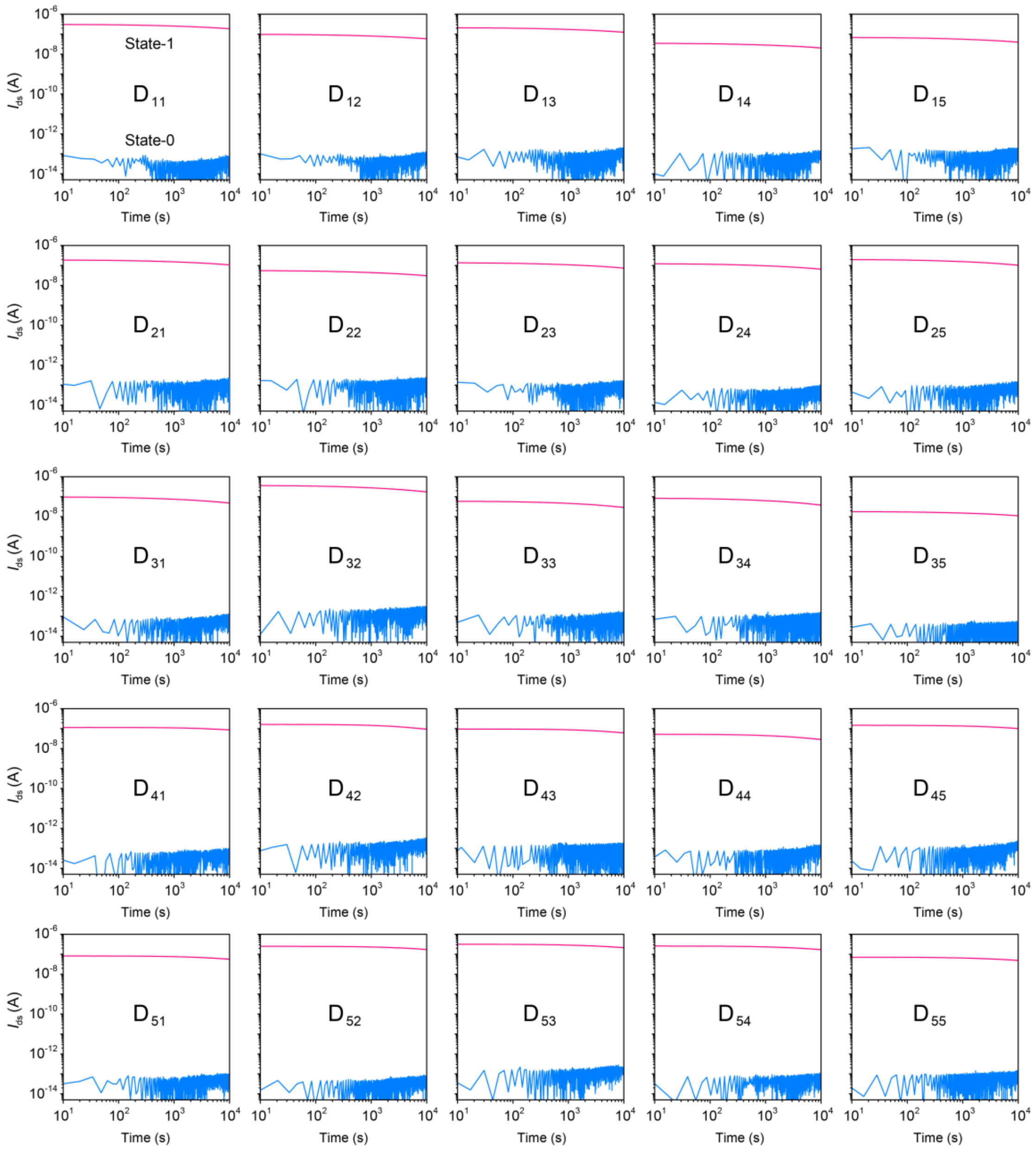

Supplementary Fig. 41| Retention characteristics of 25 memory devices based on CVD-grown MoS_2 and WSe_2 as illustrated in Supplementary Fig. 39. The readout voltage was fixed as 0.1 V.

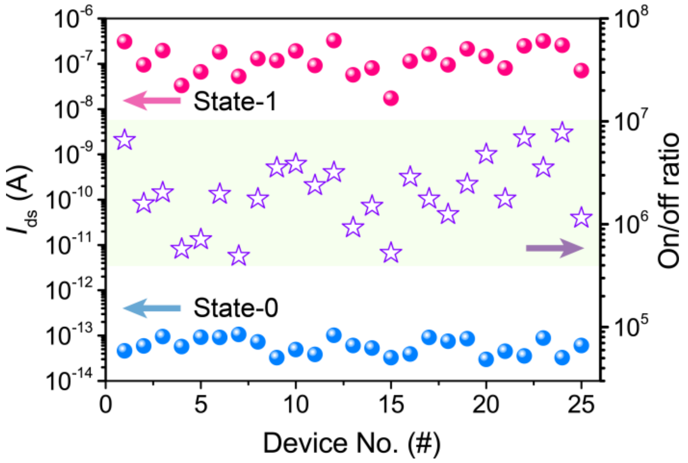

Supplementary Fig. 42| The currents of the 25 devices at State-0 and State-1 and the on/off ratio extracted from Supplementary Fig. 41.

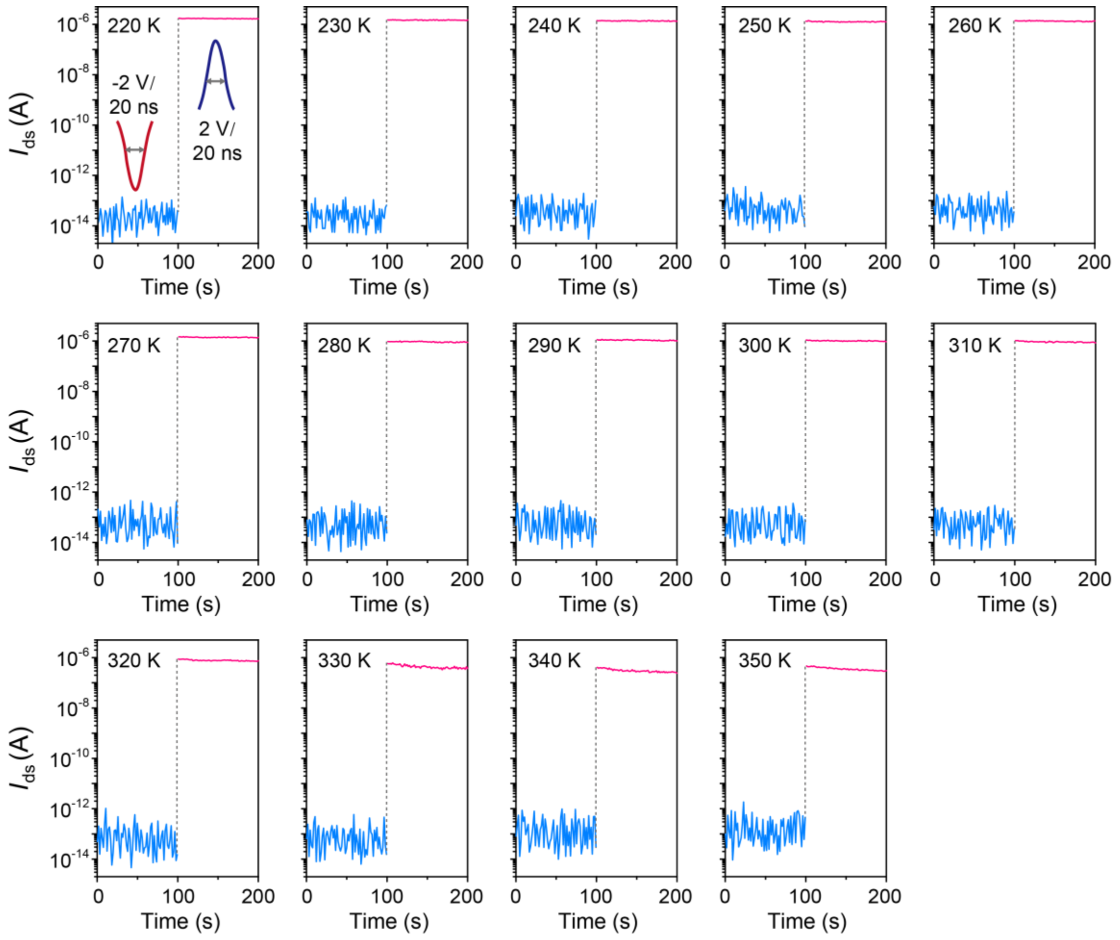

Supplementary Fig. 43| The successful reproduction of the ultrafast device performance at temperature of 220 K–350 K. 20 ns V_{CG} voltage pulses with amplitude of –2 V and +2 V were applied for the writing and erasing operations, respectively. The corresponding channel currents of state-1 and state-0 are extracted and summarized in Fig. 5a in the main text.

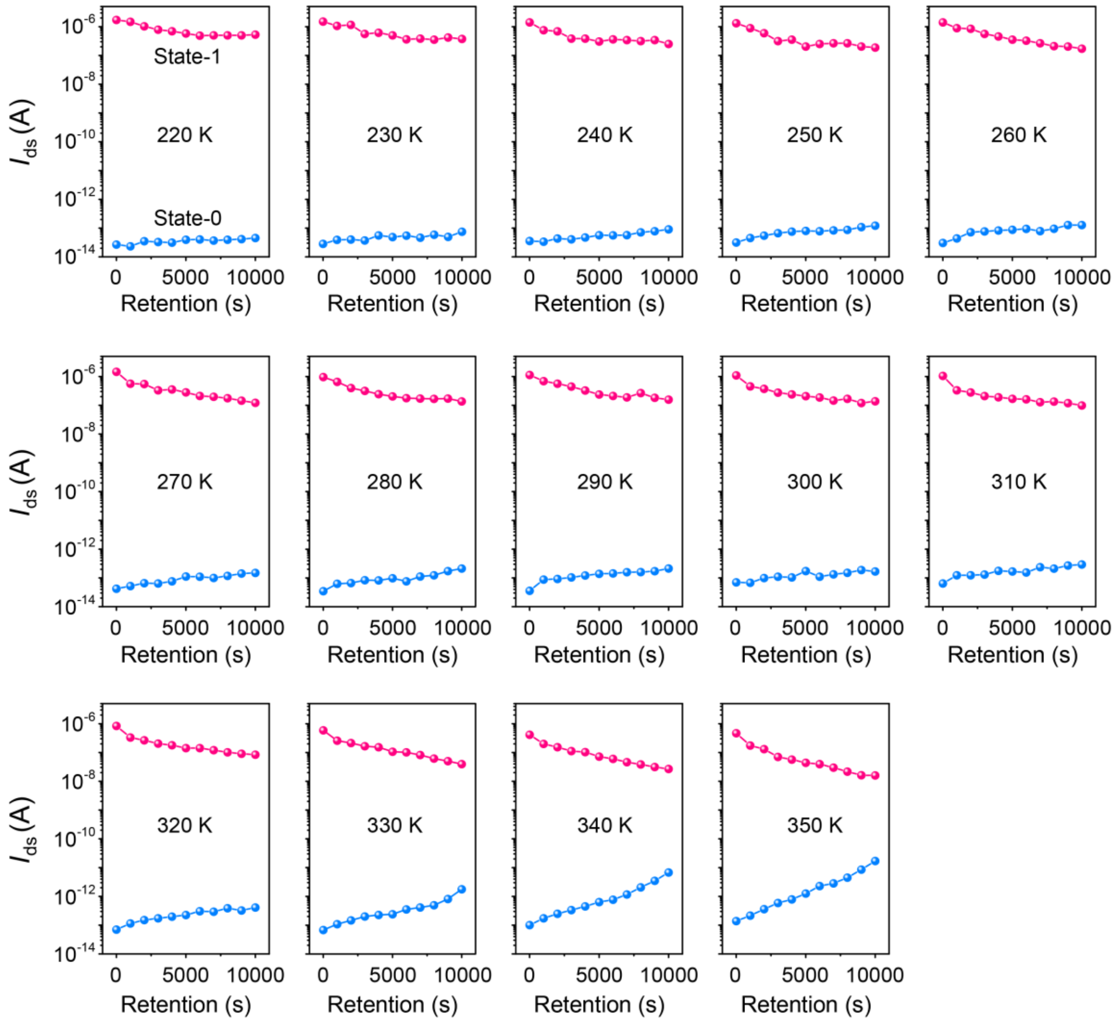

Supplementary Fig. 44| The retention characteristics of the memory device at temperature of 220 K–350 K. The corresponding channel currents of state-1 and state-0 after 10^4 s retention are extracted and summarized in Fig. 5b in the main text.

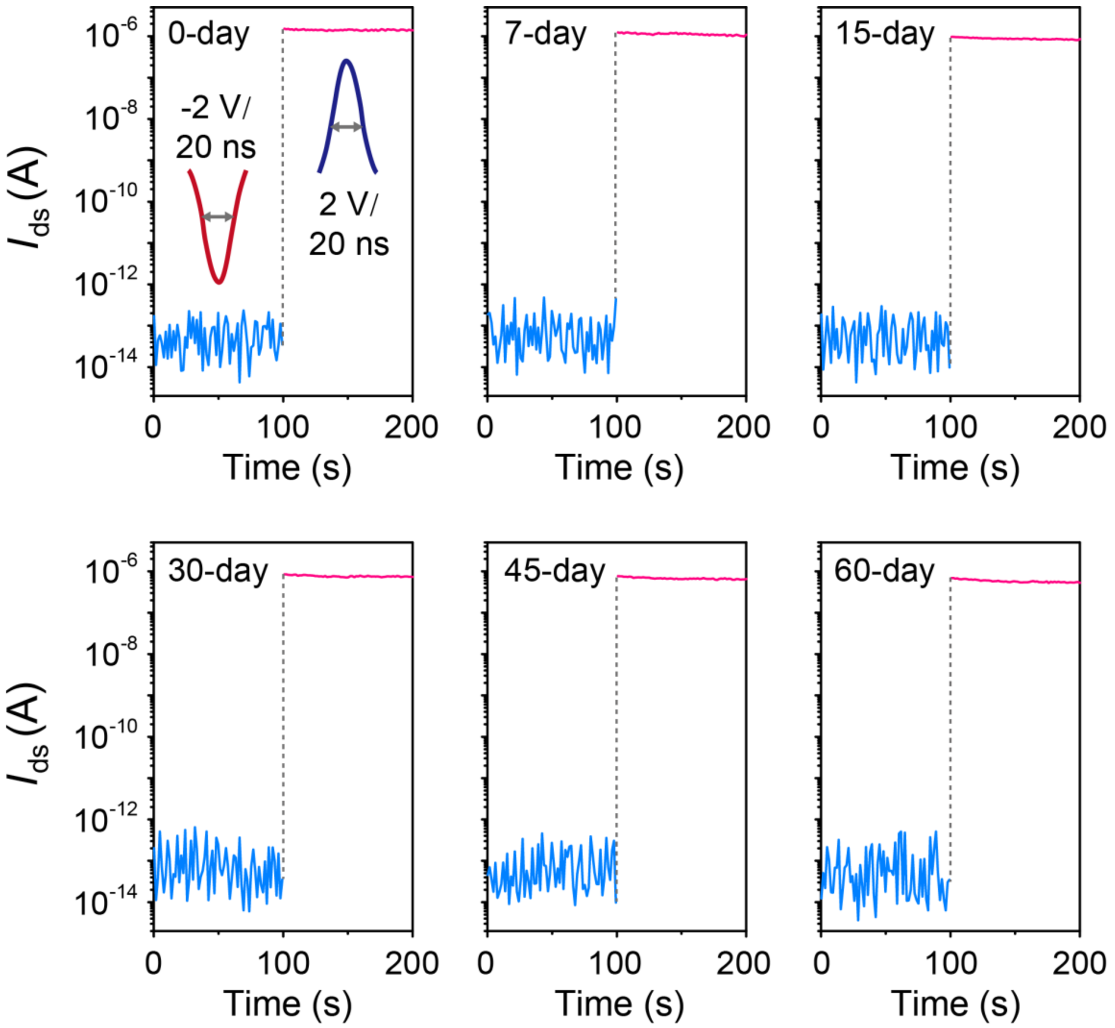

Supplementary Fig. 45| The successful reproduction of the ultrafast device performance with different preservation time after the fabrication of the memory device. 20 ns V_{CG} voltage pulses with amplitude of –2 V and +2 V were applied for the writing and erasing operations, respectively. The corresponding channel currents of state-1 and state-0 are extracted and summarized in Fig. 5c in the main text.

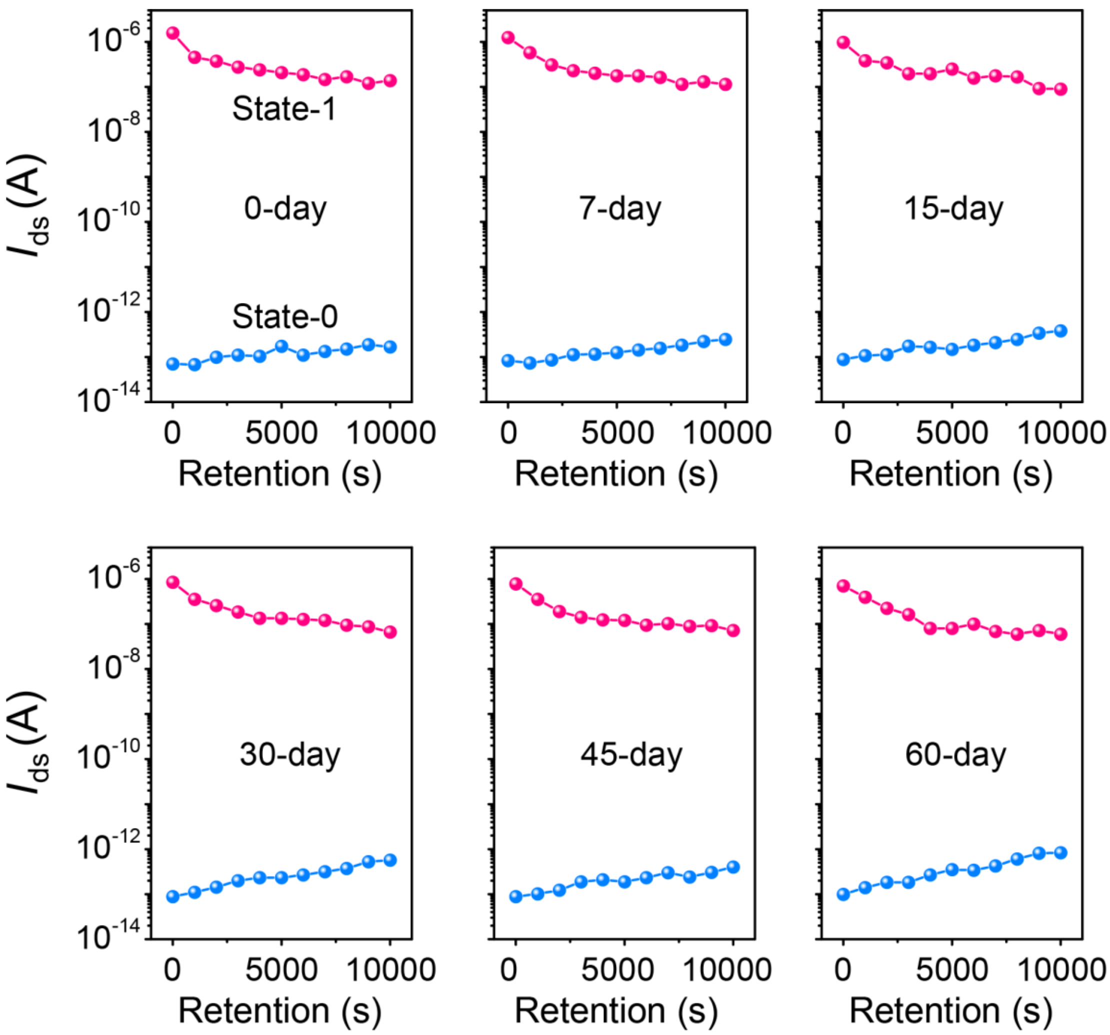

Supplementary Fig. 46| The retention characteristics of the memory device with different preservation time after the fabrication of the memory device. The corresponding channel currents of state-1 and state-0 after 10^4 s retention are extracted and summarized in Fig. 5d in the main text.

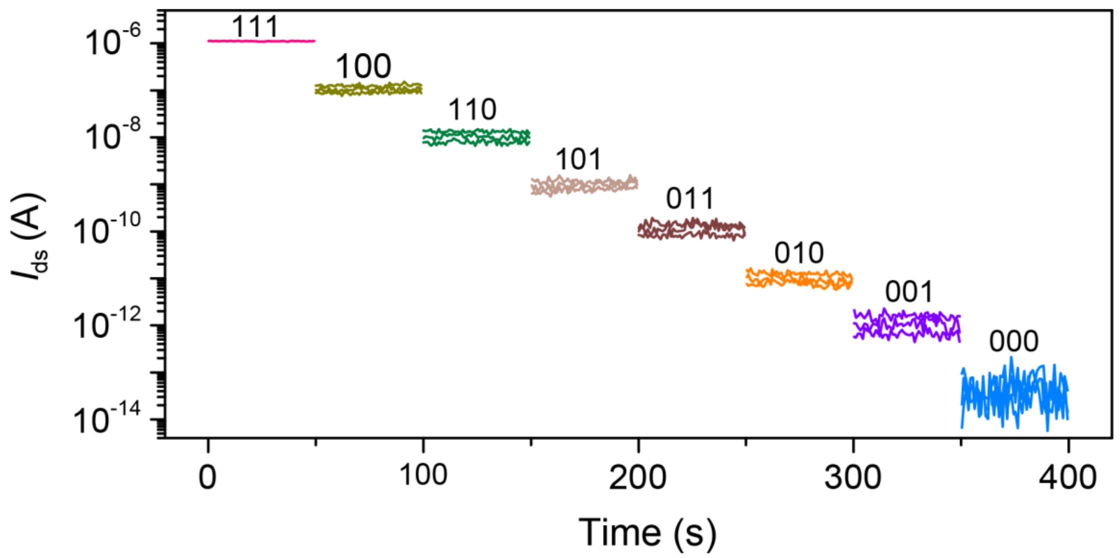

Supplementary Fig. 47| Realization of a 3-bit (8-levels) storage in the memory device. By applying a +2 V/20 ns V_{CG} pulse, the device is erased to 111 state, and the device can be programmed to different storage states (110, 101, 100, 011, 010, 001, 000) by applying 20 ns V_{CG} pulses with different amplitudes. These operations were repeated for three cycles, demonstrating excellent reliability and reproducibility.

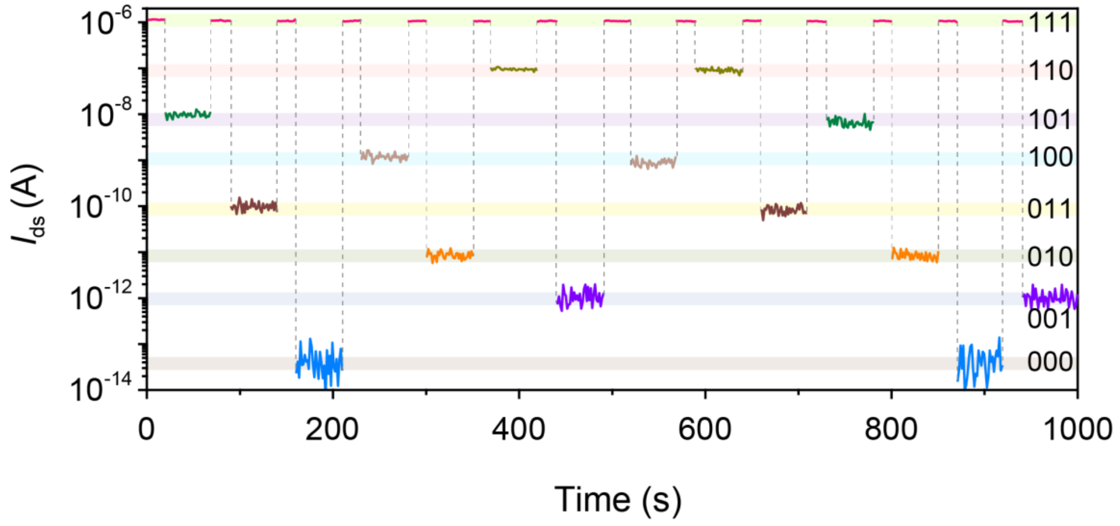

Supplementary Fig. 48| The successful access of 8 storage states with random sequence. By applying V_{CG} pulses with corresponding amplitude, each storage state can be access independently and repeatedly.

Supplementary References

1. Li, G. et al. Architecture of graphdiyne nanoscale films. *Chem. Commun.* **46**, 3256-3258 (2010).
2. Li, J. et al. Synthesis of wafer-scale ultrathin graphdiyne for flexible optoelectronic memory with over 256 storage levels. *Chem* **7**, 1284-1296 (2021).
3. Gao, L. et al. Repeated growth and bubbling transfer of graphene with millimetre-size single-crystal grains using platinum. *Nat. Commun.* **3**, 699 (2012).
4. Zhao, F. H. et al. In situ growth of graphdiyne on arbitrary substrates with a controlled-release method. *Chem. Commun.* **54**, 6004-6007 (2018).
5. Wang, H. et al. A sandwich-type photoelectrochemical sensor based on tremella-like graphdiyne as photoelectrochemical platform and graphdiyne oxide nanosheets as signal inhibitor. *Sensor. Actuat. B: Chem.* **304**, 127363 (2020).
6. Zhang, Y. et al. 2D graphdiyne oxide serves as a superior new generation of antibacterial agents. *iScience* **19**, 662-675 (2019).
7. Wu, H. et al. Interfacial charge behavior modulation in perovskite quantum dot-monolayer MoS_2 0D-2D mixed-dimensional van der Waals heterostructures. *Adv. Funct. Mater.* **28**, 1802015 (2018).
8. Fu, T. D. et al. Bioinspired bio-voltage memristors. *Nat. Commun.* **11**, 1861 (2020).
9. Wu, L. et al. Atomically sharp interface enabled ultrahigh-speed non-volatile memory devices. *Nat. Nanotechnol.* **16**, 882-887 (2021).
10. Liu, L. et al. Ultrafast non-volatile flash memory based on van der Waals heterostructures. *Nat. Nanotechnol.* **16**, 874-881 (2021).
11. Zhang, Z.-C. et al. An ultrafast nonvolatile memory with low operation voltage for high-speed and low-power applications. *Adv. Funct. Mater.* **31**, 2102571 (2021).
12. Liu, C. S. et al. A semi-floating gate memory based on van der Waals heterostructures for quasi-non-volatile applications. *Nat. Nanotechnol.* **13**, 404-410 (2018).
13. Li, J. Y. et al. Symmetric ultrafast writing and erasing speeds in quasi-nonvolatile memory via van der Waals heterostructures. *Adv. Mater.* **31**, 1808035 (2019).
14. Ding, Y. et al. A semi-floating memory with 535% enhancement of refresh time by local field modulation. *Adv. Funct. Mater.* **30**, 1908089 (2020).
15. Quoc An, V. et al. Two-terminal floating-gate memory with van der Waals heterostructures for ultrahigh on/off ratio. *Nat. Commun.* **7**, 12725 (2016).
16. Sup Choi, M. et al. Controlled charge trapping by molybdenum disulphide and graphene in ultrathin heterostructured memory devices. *Nat. Commun.* **4**, 1624 (2013).
17. Lee, D. et al. Multibit MoS_2 photoelectronic memory with ultrahigh sensitivity. *Adv. Mater.* **28**, 9196-9202 (2016).
18. Huang, W. et al. Multibit optoelectronic memory in top-floating-gated van der Waals heterostructures. *Adv. Funct. Mater.* **29**, 1902890 (2019).
19. Yang, S.-H. et al. Multifunctional full-visible-spectrum optoelectronics based on a van der Waals heterostructure. *Nano Energy* **66**, 104107 (2019).
20. Bertolazzi, S., Krasnozhon, D. & Kis, A. Nonvolatile memory cells based on MoS_2/graphene heterostructures. *ACS Nano* **7**, 3246-3252 (2013).

21. Zhang, E. et al. Tunable charge-trap memory based on few-layer MoS_2. *ACS Nano* **9**, 612-619 (2015).
22. Hou, X. et al. Charge-trap memory based on hybrid 0D quantum dot–2D WSe_2 structure. *Small* **14**, 1800319 (2018).
23. Liu, C. et al. Eliminating overerase behavior by designing energy band in high-speed charge-trap memory based on WSe_2. *Small* **13**, 1604128 (2017).
24. Wu, E. et al. Multi-level flash memory device based on stacked anisotropic ReS_2–boron nitride–graphene heterostructures. *Nanoscale* **12**, 18800-18806 (2020).
25. Kim, S. H. et al. Multilevel MoS_2 optical memory with photoresponsive top floating gates. *ACS Appl. Mater. Interfaces* **11**, 25306-25312 (2019).
26. Tian, H. et al. A dynamically reconfigurable ambipolar black phosphorus memory device. *ACS Nano* **10**, 10428-10435 (2016).
27. Kim, T. et al. 2D TMD channel transistors with ZnO nanowire gate for extended nonvolatile memory applications. *Adv. Funct. Mater.* **30**, 2004140 (2020).
28. Liu, H. N. et al. Two-dimensional WSe_2/organic acceptor hybrid nonvolatile memory devices based on interface charge trapping. *ACS Appl. Mater. Interfaces* **11**, 34424-34429 (2019).
29. Xiang, D. et al. Two-dimensional multibit optoelectronic memory with broadband spectrum distinction. *Nat. Commun.* **9**, 2966 (2018).
30. Wen, J. et al. Direct charge trapping multilevel memory with graphdiyne/ MoS_2 van der Waals heterostructure. *Adv. Sci.* **8**, 2101417 (2021).
31. Liu, X. C. et al. Charge-ferroelectric transition in ultrathin $Na_{0.5}Bi_{4.5}Ti_4O_{15}$ flakes probed via a dual-gated full van der Waals transistor. *Adv. Mater.* **32**, 2004813 (2020).
32. Huang, W. H. et al. Gate-coupling-enabled robust hysteresis for nonvolatile memory and programmable rectifier in van der Waals ferroelectric heterojunctions. *Adv. Mater.* **32**, 1908040 (2020).
